# Meta-analysis of the normal diffusion tensor imaging values of the median nerve and how they change in carpal tunnel syndrome

**DOI:** 10.1038/s41598-021-00353-z

**Published:** 2021-10-22

**Authors:** Djamila Rojoa, Firas Raheman, Joseph Rassam, Ryckie G. Wade

**Affiliations:** 1 Department of Plastic and Reconstructive Surgery, Leicester Royal Infirmary, Leicester, UK; 2 Department of Plastic and Reconstructive Surgery, Leeds Teaching Hospitals Trust, Leeds, UK; 3 Leeds Institute for Medical Research, Advanced Imaging Centre, University of Leeds, Leeds, LS1 3EX UK

**Keywords:** Neurological disorders, Peripheral neuropathies, Magnetic resonance imaging

## Abstract

Carpal tunnel syndrome (CTS) leads to distortion of axonal architecture, demyelination and fibrosis within the median nerve. Diffusion tensor imaging (DTI) characterises tissue microstructure and generates reproducible proxy measures of nerve ‘health’ which are sensitive to myelination, axon diameter, fiber density and organisation. This meta-analysis summarises the normal DTI values of the median nerve, and how they change in CTS. This systematic review included studies reporting DTI of the median nerve at the level of the wrist in adults. The primary outcome was to determine the normal fractional anisotropy (FA) and mean diffusivity (MD) of the median nerve. Secondarily, we show how the FA and MD differ between asymptomatic adults and patients with CTS, and how these differences are independent of the acquisition methods. We included 32 studies of 2643 wrists, belonging to 1575 asymptomatic adults and 1068 patients with CTS. The normal FA was 0.58 (95% CI 0.56, 0.59) and the normal MD was 1.138 × 10^–3^ mm^2^/s (95% CI 1.101, 1.174). Patients with CTS had a significantly lower FA than controls (mean difference 0.12 [95% CI 0.09, 0.16]). Similarly, the median nerve of patients with CTS had a significantly higher mean diffusivity (mean difference 0.16 × 10^–3^ mm^2^/s [95% CI 0.05, 0.27]). The differences were independent of experimental factors. We provide summary estimates of the normal FA and MD of the median nerve in asymptomatic adults. Furthermore, we show that diffusion throughout the length of the median nerve becomes more isotropic in patients with CTS.

## Introduction

Carpal tunnel syndrome (CTS) is the most common compressive neuropathy, affecting 10 million people annually. Consequently, CTS is the most expensive upper extremity musculoskeletal disorder, costing the USA health system over $2 billion annually and employers up to $114,000 per incident^1^.

**Figure 1:**
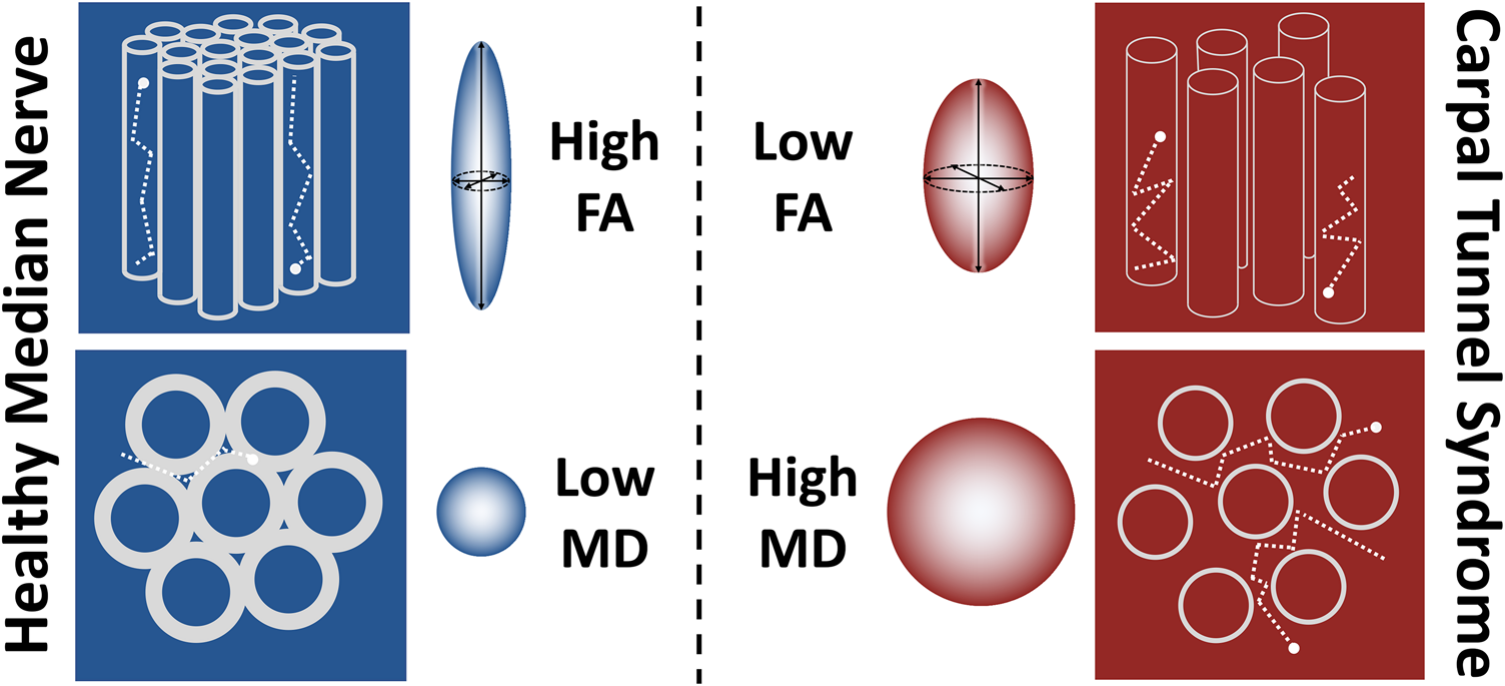
A diagram of nerve fibres (top) and in cross-section (bottom) demonstrating how diffusion tensor imaging metrics change in CTS. In healthy nerves, the axons are enveloped by myelin sheaths and arranged relatively tightly, which restricts the diffusion of water to the long axis of the nerve. Chronic compression leads to distortion of the axonal architecture, demyelination and as such, degradation of physiological barriers to the diffusion of water diffusion. Consequently, more diffusion occurs perpendicular to the long axis of the nerve as water is more free to diffuse around the fibres, reducing the factional anisotropy (FA) and increasing the magnitude of diffusion (mean diffusivity, MD).

Compression of peripheral nerves leads to distortion of the axonal architecture, demyelination with or without poor remyelination, loss of the intrinsic vasculature and ultimately, fibrosis of the perineurial and epineurial connective tissue^2,3^. Diffusion tensor imaging (DTI) characterises tissue microstructure and generates reproducible^4–8^ proxy measures of nerve ‘health’ which are sensitive to myelination, axon diameter, fibre density and organisation^9–11^ (Fig. 1). DTI typically generates the following metrics: fractional anisotropy (FA), mean diffusivity (MD), axial diffusivity (AD) and radial diffusivity (RD). FA is a scalar value between zero and one—a FA of zero implies isotropic diffusion of water within a voxel, whilst a FA of one implies diffusion along a single axis (i.e., bidirectional diffusion along the length of the nerve). MD describes the average molecular diffusion rate within the voxel, whilst AD describes diffusion in the long axis and RD represents diffusion perpendicular to the long axis.

Several studies have shown that DTI metrics (FA and MD) are sensitive to microstructural changes which occur within the median nerve of patients with CTS (Fig. 1). However, there are several uncertainties that must be resolved before this technology could be used in clinical practice or as a reference standard in research studies. Firstly, the normal DTI values of the median nerve must be established and secondly, uncertainty around how experimental conditions (e.g. scanning parameters) influence DTI metrics need to be determined. These uncertainties, and how DTI metrics change in CTS, might be resolved through meta-analysis and represents the rationale for this study.

## Methods

This review is registered with PROSPERO (CRD42020212378). It was designed and conducted in accordance with the Cochrane Handbook of Systematic Reviews^12^ and has been authored in accordance with the PRISMA 2020 statement^13^.

### Types of studies

We included all studies which reported the findings of diffusion tensor magnetic resonance imaging of the median nerve, at the level of the wrist in asymptomatic adults or adults with CTS. There were no language restrictions. We excluded case reports and studies which did not report DTI metrics (e.g., studies which contained fibre tractography graphics only) of the median nerve.

### Participants

This review considers 2 distinct populations:

1. Asymptomatic adults (aged ≥ 16 years) with no known pathology (past or present) affecting the peripheral nerves of the upper limb.
2. Adults with a diagnosis of carpal tunnel syndrome. For a study to be included, we did not impose any specific thresholds or criteria on the diagnosis of CTS, such as the presence of specific symptoms, provocative tests, aberrant electrophysiological parameters or imaging features.

### Search strategy

The NICE Healthcare Databases (hdas.nice.org.uk) was searched according to Appendix 1 (Supplementary Materials) on 9th October 2020. The medRxiv and bioRxiv preprint archives were searched with the same strategy using the R package medrxivr^14^. Later, CitationChaser^15^ was used for forward and backward citation chasing based on the final list of included studies (eFigure 1).

### Study selection

Three review authors (DR, JR and FR) independently screened titles and abstracts for relevance, in accordance with the eligibility criteria. The full texts of potentially eligible articles were obtained and again independently assessed by the same authors. Disagreements were resolved by discussion with RGW. The reasons for excluding studies are outlined in Appendix 2 (Supplementary Materials).

### Data extraction

Three review authors (DR, JR and FR) independently double extracted all data. Thereafter, all datapoints were independently checked for accuracy by RGW. DTI parameters were extracted from the following anatomical levels of the median nerve: the distal radio-ulnar joint (DRUJ), the pisiform and the hook of the hamate, as these are three commonly used imaging landmarks which equate to the inlet, mid-point and outlet of the carpal tunnel. The nerve/hand was the unit of analysis^16^. Many studies reported both the number of individuals and wrists scanned (as some studies involved bilateral scanning) but if not otherwise stated we assumed imaging was performed unilaterally. If data were missing, unclear or present in an unfavourable format then the authors were contacted by email with a request for more information. Four authors provided additional information upon request^17–20^. When no reply was received, estimates were derived from graphs or imputed where possible^21^.

### Outcomes

The primary outcome was to estimate the normal DTI metrics (FA and MD) of the median nerve in asymptomatic adults. The secondary outcomes were to estimate the differences in DTI metrics (FA and MD) between asymptomatic adults and patients with CTS, and explore the associations between DTI metrics and: age, echo time (TE), repetition time (TR), resolution, the number of diffusion sensitising gradient directions (N_D_) sampled per shell, the b-value(s), different methods of k-space sampling and in-plane acceleration.

### Methodological quality assessment

The risk of bias was independently assessed by three review authors (DR, JR and FR) using the ROBINS-I tool^22^ and displayed graphically using robvis^23^. Disagreements were resolved by discussion with RGW.

### Statistical analysis

The raw data are available via the Open Science Framework (https://osf.io/vqwkp/). The single study performed at 7 tesla^19^ was excluded from all meta-analyses given its clinical disparity. Using the *meta* suite of Stata v16 (StataCorp, Texas), the mean FA and MD from asymptomatic adults were pooled to estimate the normative values. We performed direct comparisons meta-analysis of the mean differences in FA and MD between asymptomatic adults and patients with CTS. Meta-analyses were subgrouped by anatomical location. Restricted maximum likelihood was used to estimate the between-study variance (tau^2^), with the Knapp and Hartung modification. Heterogeneity was quantified by I^2^ ^24^.Using the metafor^25^ package, mixed-effects meta-regression was used to explore potential reasons for the observed heterogeneity in the direct comparisons meta-analysis of FA; the continuous covariates were age, in-plane resolution (mm^2^), slice thickness (mm), echo time (TE in ms), b-value (mm^2^/s) and number of diffusion-sensitising gradient directions (N_D_). TE and b-value were modelled as an interaction. Confidence intervals (CI) were generated to the 95% level. To investigate the possibility of small-study effects for FA between asymptomatic adults and patients with CTS, a funnel plot was constructed with the pseudo CIs contoured by tau^2^ ^25^. EviAtlas was used to generate a map of the location of the 1st author’s institution^26^.

## Results

Ultimately, 32 studies^9,17–20,27–53^ were included (eFigure 2).

### Study characteristics

Study characteristics are detailed in eTable 1. Overall, we included data from 2643 wrists belonging to 1575 asymptomatic adults and 1068 patients with CTS. Asymptomatic adults were a mean 6 years younger than patients with CTS (95% CI 3, 10). There were approximately twice as many females (1404:746) although this disparity was more pronounced in patients with CTS (660F:193 M) than asymptomatic adults (737F:552 M). The median number of authors was 6 (IQR 5–8) and studies were derived from 16 different countries (eFigure 3).

Ten studies (32%) were performed at a field strength of 1·5 tesla^18,30, 33,36,39,43,45,49,50,52^, twenty one (65%) at 3 tesla^9,17,20,27–29,31,32,34,35,37,38,40–42,44,46–48,51,53^, and one at 7 tesla^19^. The median echo and repetition times were 87 ms (IQR 65–91, range 21–103) and 7000 ms (IQR 3800–7650, range 1470–10,254), respectively. Two studies used read-out segment echo-planar imaging (rsEPI)^34,46^, two did not specify^20,48^ and the remainder used single-shot echo-planar imaging (ssEPI). Twelve studies described in-plane acceleration techniques (GRAPPA^19,28,42^, SENSE^18,29,32,39,41,46,51^ and CAIPIRINHA^31^) and six studies used partial Fourier transformations^31,33,35,39,41,44,46^. The median slice thickness was 3·5 mm (IQR 2·6–4·0 mm, range 1·5–5 mm). The median in-plane resolution was 1·09 mm (IQR 0·7–1·5, range 0·4–1·88). Two studies investigated multiple b-values^36,51^ via discrete shells, although no studies reported whether acquisitions were half or whole shell and what sample scheme was used. The mean b-value was 1000 s/mm^2^ (SD 270, range 325–2000). The median N_D_ was 20 (IQR 15–25, range 6–32). A median of 3 signal averages (excitations) were obtained (IQR 2–5, range 1–12). When reported, the mean SNR of the b0 images was 25 (SD 12)^19,29,31,33,36,39^.

The risk of bias for the included studies is summarised in eFigure 4. The majority of studies were at low risk of methodological bias.

### Evidence synthesis: asymptomatic adults

The FA of the median nerve in asymptomatic adults was reported in 29 studies^9,17–20,27–36,38–42,44–51,53^. Overall, the normal FA was 0.58 (95% CI 0·56, 0.59; I^2^ 98%). The FA was highest at the level of the DRUJ (mean 0.61 [95% CI 0.58, 0.63]; I^2^ 96%), dropping at the level of the pisiform to 0.57 (95% CI 0.54, 0.61; I^2^ 98%) and lowest at the level of the hook of the hamate (mean 0.54 [95% CI 0.51, 0.57]; I^2^ 95%).

The MD of the median nerve in asymptomatic adults was reported in 28 studies^9,17–20,27–36,38–42,44–51^. Overall, the normal MD was 1·138 × 10^–3^ mm^2^/s (95% CI 1.101, 1.174; I^2^ 99%). The MD was lowest at the level of the DRUJ (mean 1.073 × 10^–3^ mm^2^/s [95% CI 1.019, 1.128]; I^2^ 93%), increasing at the level of the pisiform (mean 1.180 × 10^–3^ mm^2^/s [95% CI 1.115, 1.244]; I^2^ 96%) and highest at the level of the hook of the hamate (mean 1.151 × 10^–3^ mm^2^/s [95% CI 1.086, 1.217]; I^2^ 98%).

### Evidence synthesis: patients with CTS

The FA of the median nerve in patients with CTS was reported in 19 studies^17–20,27–29,32–34,38–40,45,47–50,52^. Overall, patients had a mean FA of 0.45 (95% CI 0.43, 0.47; I^2^ 95%). The FA was lowest at the mid-point of carpal tunnel, at the level of the pisiform (mean 0.41 [95% CI 0.38, 0.43]; I^2^ 86%), compared to the levels of the DRUJ (mean 0.48 [95% CI 0.44, 0.52]; I^2^ 91%) or hook of the hamate (mean 0.45 [95% CI 0.42–0.48]; I^2^ 93%).

The MD of the median nerve in patients with CTS was reported in 18 studies^17–20,27–29,32–34,39,40,45,47–50,52^. Overall, patients with CTS had a pooled mean MD of 1.293 × 10^–3^ mm^2^/s (95% CI 1.227, 1.359; I^2^ 99%). The MD was highest at the level of the pisiform (mean 1.372 × 10^–3^ mm^2^/s [95% CI 1.245–1.500]; I^2^ 98%), 1.180 × 10^–3^ mm^2^/s at the level of the DRUJ (95% CI 1.064, 1.295; I^2^ 95%) and 1.335 × 10^–3^ mm^2^/s at the level of the hook of the hamate (95% CI 1.259, 1.411; I^2^ 93%).

### Direct comparisons meta-analysis: asymptomatic adults vs. patients with CTS

**Figure 2:**
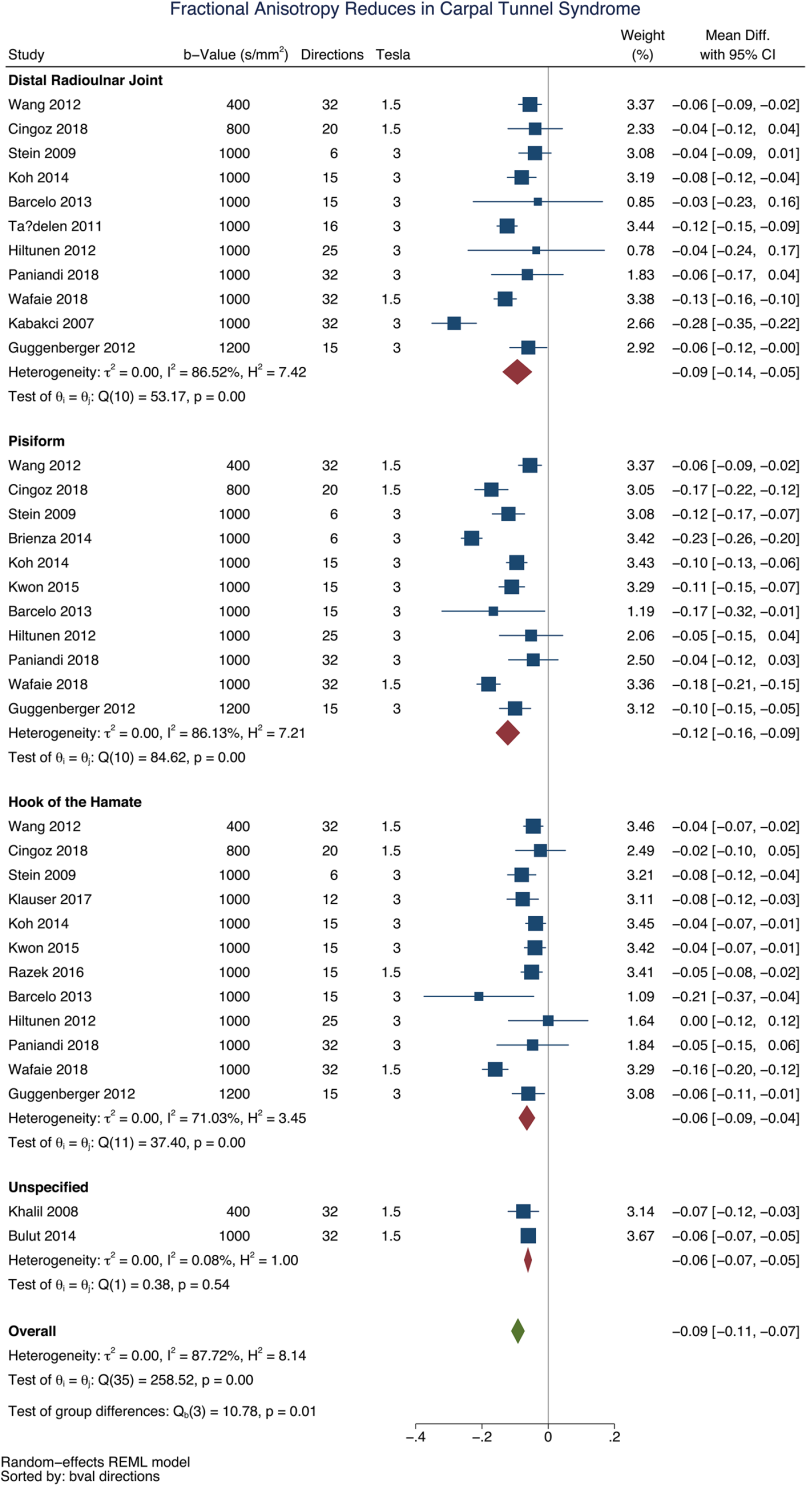
A forest plot of the fractional anisotropy of the median nerve, at 3 anatomical levels, between asymptomatic adults and patients with carpal tunnel syndrome.

Fourteen studies reported direct comparisons between asymptomatic adults and patients with CTS^17–19,28,29,32–34,39,40,45,47,49,50^. All studies reported a lower FA in patients with CTS compared to asymptomatic adults (mean difference 0.09 [95% CI 0.07, 0.11]; Fig. 2). The largest difference between asymptomatic adults and patients with CTS was at the mid-point of the carpal tunnel, at the level of the pisiform (mean difference 0.12 [95% CI 0.09, 0.16]).

**Figure 3:**
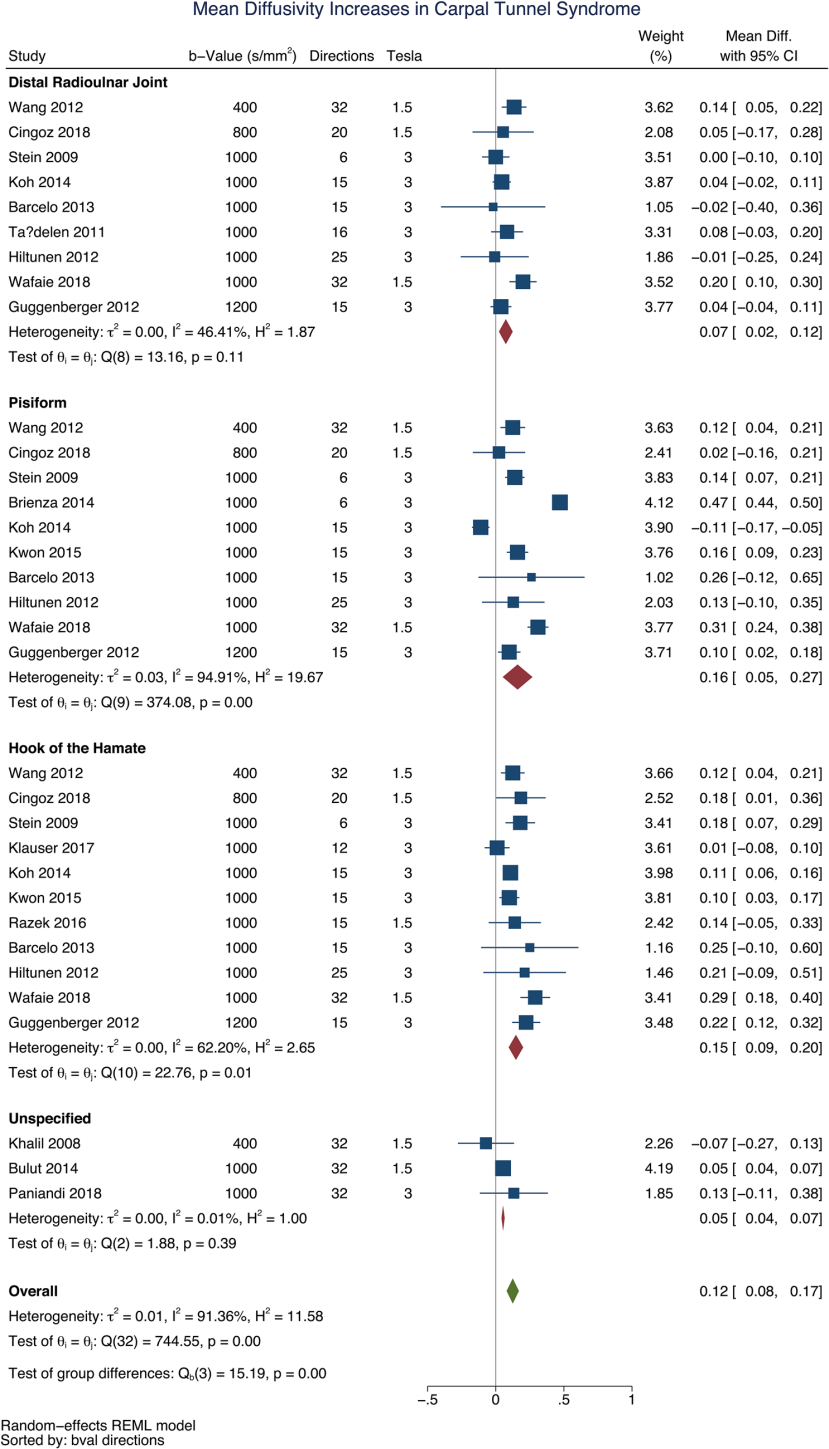
A forest plot of the mean diffusivity of the median nerve, at 3 anatomical levels, between asymptomatic adults and patients with carpal tunnel syndrome.

Patients with CTS had a higher mean diffusivity than asymptomatic adults (mean difference 0.12 × 10^–3^ mm^2^/s [95% CI 0.08, 0.17], Fig. 3). This difference was again most profound at the mid-point of the carpal tunnel, at the level of the pisiform (mean difference 0.16 × 10^–3^ mm^2^/s [95% CI 0.05, 0.27]).

### Meta-regression

**Figure 4:**
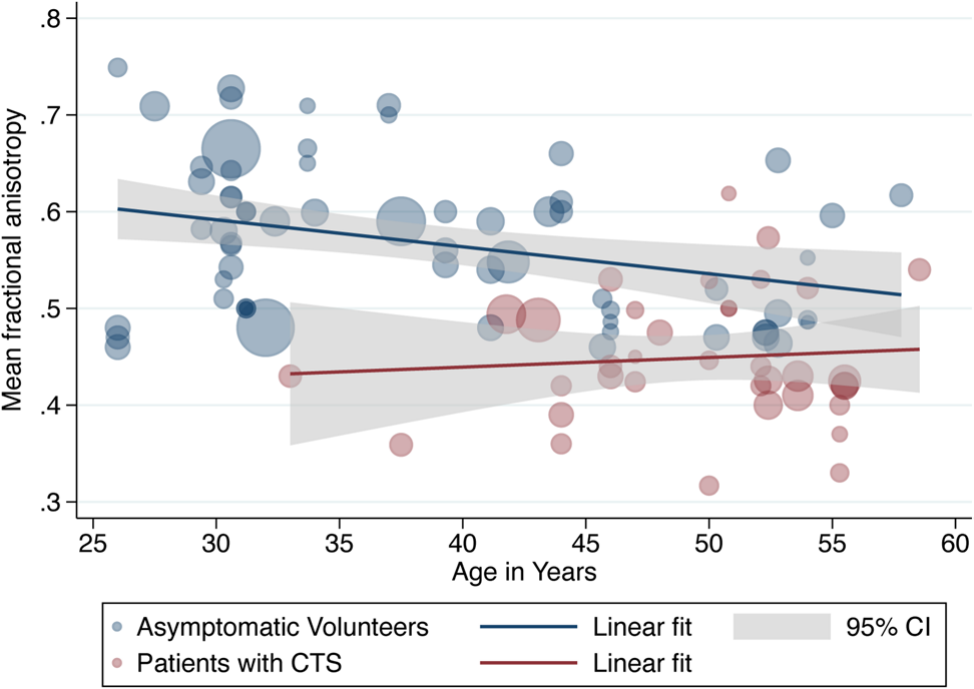
A scatterplot of study-level estimates of fractional anisotropy in asymptomatic adults and patients with carpal tunnel syndrome, against age in years. The size of the points corresponds to the precision (inverse variance) of the study.

**Figure 5:**
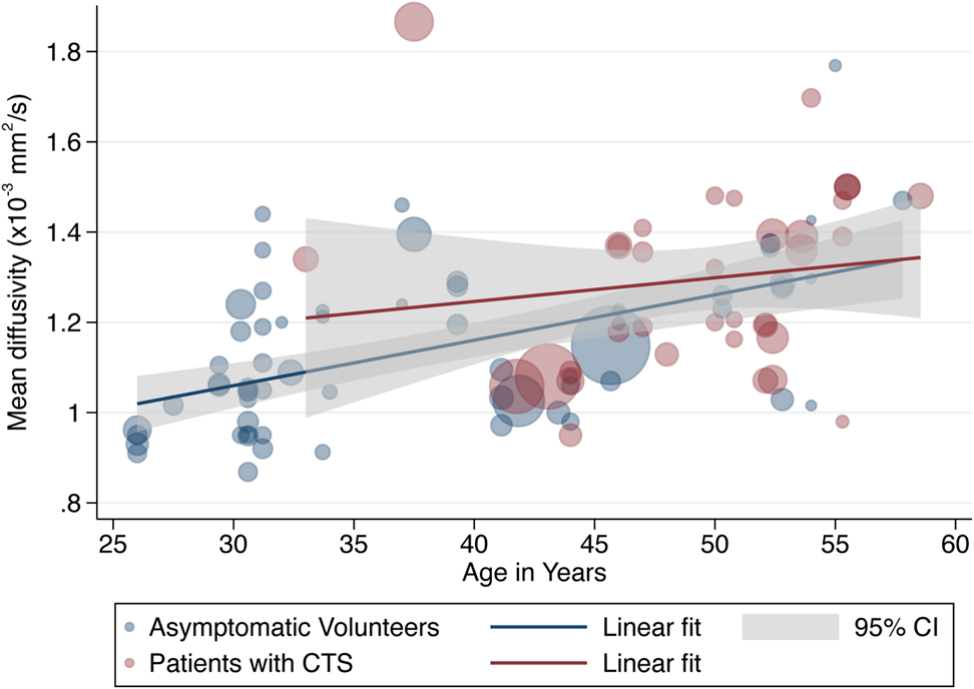
A scatterplot of study-level estimates of mean diffusivity in asymptomatic adults and patients with carpal tunnel syndrome, against age in years. The size of the points corresponds to the precision (inverse variance) of the study.

Age was negatively associated with the FA in asymptomatic adults whereby each decade of life reduced the FA by approximately 0·003 (adjusted β – 2.79 × 10^–3^ [95% CI – 4.78 × 10^–3^, − 8.12 × 10^–4^]; I^2^ 97%). However, there was no relationship between age and FA in patients with CTS (adjusted β 9.70 × 10^–4^ [95% CI – 2.89 × 10^–3^, 4.83 × 10^–3^; I^2^ 96%], Fig. 4). Increasing age was also associated with MD whereby each decade of life increased MD by approximately 0.108 × 10^–4^ mm^2^/s (95% CI 0.073 × 10^–4^, 0.140 × 10^–4^; I^2^ 99%, Fig. 5) with no significant difference between asymptomatic adults and patients with CTS.

There was no relationship between N_D_ and FA (eFigure 5) or MD (eFigure 66). The b-value was not associated with the FA (eFigure 7). There was an inverse relationship between the b-value and MD, whereby increments of 100 mm^2^/s reduced the mean diffusivity by 0.04 × 10^–3^ mm^2^/s (β − 3.849 × 10^–7^ mm^2^/s [95% CI − 5.019 × 10^–7^, − 2.678 × 10^–7^]; I^2^ 98%, eFigure 8).

There were no significant differences between studies which used ssEPI or rsEPI. There was no association between the in-plane resolution (in square millimetres) and FA (eFigure 9) or MD (eFigure 10). Slice thickness was not associated with FA (eFigure 11) but was negatively associated with MD whereby increments of 1 mm reduced the MD by 6.023 × 10^–5^ mm^2^/s (95% CI 9.754 × 10^–5^, 2.294 × 10^–5^; I^2^ 99%, eFigure 12).

The TE was not associated with FA or MD (eFigures 13 and 14). The TR was not associated with FA (eFigure 15) but longer repetition times were associated with lower estimates of MD, whereby increasing the TR by 1 s decreased the MD by 2.990 × 10^–6^ mm^2^/s (95% CI 4.383 × 10^–6^, 1.598 × 10^–6^; eFigure 16).

Studies reporting the use of parallel imaging techniques (e.g. GRAPPA, SENSE or ASSET) yielded 5% higher estimates of FA (β 0.05 [95% CI 0.02, 0.08]; I^2^ 98%, eFigure 17) when compared to studies which did not report this information. Parallel imaging methods were not associated with differences in the MD. There was insufficient data to explore different partial Fourier settings. There was no association between the number of signal averages and FA or MD (eFigures 18 and 19).

Ultimately, mixed-effects multivariable meta-regression showed that having CTS was the strongest independent moderator of the observed heterogeneity in FA (Table 1). Age explained some of the residual between-study variance. The experimental factors we modelled did not explain the residual heterogeneity.

**Table 1:** Mixed-effects meta-regression.

Covariates	Adjusted change in fractional anisotropy (β)	95% CI	Resampled p-value
Patients with carpal tunnel syndrome	− 8.57 × 10^–2^	− 0.13, − 0.06	0.000
Age in years	− 2.92 × 10^–3^	− 4.54 × 10^–3^, − 7.34 × 10^–4^	0.007
Repetition time (ms)	− 8.98 × 10^–6^	− 1.14 × 10^–5^, 6.77 × 10^–8^	0.053
Number of diffusion sensitising gradient directions	2.27 × 10^–3^	− 8.39 × 10^–4^, 2.99 × 10^–3^	0.271
Echo time (ms) and b-value (mm^2^/s)*	− 1.51 × 10^–8^	− 5.27 × 10^–7^, 4.86 × 10^–7^	0.936

Adjusted R^2^ = 46%, tau^2^ = 0·0047, I^2^ = 97%.

*Echo time is a function of the b-value as larger b-values mandate relatively longer echo times, so these variables are modelled as the product to minimise the number of covariables, mitigate collinearity and model the interaction between the two variables.

There was no evidence of publication bias (Eggers β 0.10 [95% CI 0.06, 0.14]; p = 0.134, eFigure 20).

## Discussion

This study demonstrates that throughout the length of the median nerve at the wrist, patients with CTS have more isotropic diffusion than asymptomatic adults. The largest differences for both fractional anisotropy and mean diffusivity were observed at the mid-point of the carpal tunnel, at the level of pisiform where CTS patients had lower FA and higher MD. Of clinical importance, we demonstrate that these real-world differences were independent of age and experimental (acquisition) conditions. Therefore, we believe that aberrations in both FA and diffusivity could be used to identify patients with median nerve neuropathy at the wrist.

There are inherent problems with clinicians diagnosing CTS given that the constellation of symptoms and clinical signs of the syndrome, and the available tests are largely unreliable. For example, nocturnal paraesthesias and many classical tests such as Phalen and Tinel, the scratch-collapse and sensory threshold testing have poor diagnostic value^54,55^. Despite the widespread use of electrodiagnostic studies in patients with suspected carpal tunnel syndrome, it remains an invasive test which evokes pain and anxiety, and controversy still exists regarding its accuracy and the normal values^56,57^. For these reasons, surgeons still perform decompression surgery in the presence of normal provocative and electrodiagnostic tests^58^. More recently, measurement of the cross sectional area of the median nerve using ultrasound is gaining popularity given that it has good inter-rater and intra-rater reliability^59^, and validity^60,61^. Despite these benefits, ultrasound has not been adopted into routine clinical practice because several aspects remain unclear, such as (i) how the cross-sectional area is affected by other factors such as age, sex, diabetes, sonographer technique [pressure applied, measurement angle, etc.] and the hardware, (ii) whether the epineurium should be included in the measurement, and (iii) how these measurements relate to severity, subjective and objective outcomes. Moreover, sonographically derived cross-sectional area still only provides morphological information (size and shape) which is inherently insensitive to nerve function and microstructure. Therefore, considerable effort has been directed towards the development of DTI because it characterises tissue microstructure and generates reproducible^4–8^ proxy measures of nerve ‘health’ which are sensitive to myelination, axon diameter, fibre density and organisation^9–11^ (Fig. 1). DTI metrics outperform standard morphological imaging in patients with peripheral neuropathy^35^ and are independent of age and acquisition parameters, something which cannot be said for electrodiagnostics^62^ or sonography. In the UK, the cost of a non-contrast MRI of the extremity is less than an electrodiagnostic exam (£389 versus £444) but more than sonography (£220)^63^. And DTI would be supplemented with other MRI data, such as morphological (anatomical) scans, contrast-free angiography, sequences which characterise muscle (fat fractions, elastography, iron deposition, etc.) and the topography of the sensorimotor cortex (e.g., using functional MRI) to determine whether there is central capacity to regenerate following peripheral nerve surgery. Ultimately, we have not examined the diagnostic test accuracy of each modality head-to-head and this must be performed before comments about relatively accuracy and cost effectiveness can be made. Overall, we suggest that DTI might provide additional valuable information for the diagnosis, grading and management of (at least unclear or complex) patients with CTS. However, incorporating DTI in the real-world management of CTS would be difficult and require significant training for clinicians, changes to infrastructure and clinical pathways. None-the-less, we show that DTI yields unique information about the ‘health’ of the median nerve which could of significant clinical value. Initially, this technology could be used in patients with an unclear diagnosis or bilateral symptomatology, and those who don’t improve after treatment.

We observed high statistical heterogeneity which has many potential explanations. The majority of the (statistical) heterogeneity was explained by the presence of CTS and it is plausible that the remainder is explained by the ‘severity’ of disease, which we were unable to capture. For example, we speculate that patients with more severe CTS (e.g., symptoms for years, resulting in profound demyelination, axonal loss and fibrosis) are likely to have lower FA and higher MD than patients with recent-onset mild CTS. Age also explained some of the observed heterogeneity and this is unsurprising, given that FA is known to fall in aging peripheral nerves^64^, just as it does in the white matter tracts of the brain^65,66^. This is because aging axons lose integrity, undergo demyelination and there is a simultaneous increase in extra-cellular fluid. Importantly, we showed that DTI metrics were sensitive to CTS after adjusting for age. Finally, in highly controlled and extreme conditions, user-specified factors^67^ such as the SNR^68^, b-value^69,70^, N_D_^71,72^, distortion correction pipelines^73,74^, tensor fitting methods^68^ and partial volume effects^75^ have been shown affect the DTI parameter estimates, which may explain some of the remaining heterogeneity. Although we could not explore the effects of all these factors, in general we observed that experimental conditions had little or no significant effect on the measured FA or MD. Therefore, despite the statistical heterogeneity, DTI appears to be reliably sensitive to the microstructural changes of the median nerve which occur in CTS.

There were no significant associations between FA and MD, and several core elements of the pulse sequence. Therefore, we suggest that clinicians and researchers wishing to acquire DTI could optimise their sequence as follows. As tensors are robust to varying b-values (in the hindered range) we suggest a b-value of 300-800 mm^2^/s; smaller b-values enable a shorter TE, which improves SNR and mitigates the effects of T2 shine-through at the expense of less diffusion-weighting. Reducing the TE might also enable users to take advantage of other vendor-specific options to improve data quality and reduce distortions. Given that the median nerve has no crossing fibres to model, it is not tortuous (within or between voxels) and increasing the N_D_ has little effect on simple tensor fitting^76^, we see no reason for the N_D_ to exceed approximately 15. The normal median nerve has a cross-sectional area of 9 mm^2^ (3.4 mm diameter)^77^ and this increases with CTS^78^, so we recommend an in-plane resolution of approximately 1 mm^2^ to ensure that at least 1 voxel is not affected by partial voluming^75^. As that the median nerve is orthogonal to the imaging plane (if data are acquired axially), the slice thickness could be comfortably increased until there is adequate SNR because it appears to have little effect on the resultant metrics. Until work is published to show the exact relaxation properties (T1, T2, T2*) of the median nerve we suggest that TR is set to approximately 4500 ms to reduce scan time. Ideally, users specify an even number of signal averages (full datasets), divided equally between opposing phase-encoding directions (i.e. 1 signal acquisition blip-up and 1 blip-down or 2 averages blip-up and 2 blip-down, etc.) as this would allow offline concatenation and exploitation of the various corrections available in the FMRIB Software Library (FSL)^79^. We advocate capturing such data via ssEPI because it is more time efficient than current implementations of rsEPI and distortions associated with ssEPI can be ameliorated using various softwares. It should be noted that thicker slices, longer repetition times and more signal averages are associated with lower estimates of MD, if this is of importance to users.

### Limitations

The main limitation of this study is the inherent and pervasive problem of CTS diagnosis which may have biased the findings. At present there is no internationally agreed diagnostic criteria for CTS and as such, there is clinical variation which is present in the includes studies. We planned to capture disease severity from the original studies, but this information was not available. As a matter of urgency, the community should work towards a consensus on objective criteria which constitute a diagnosis of CTS. Thereaftere, a reference standard for the diagnosis can be defined and this would enable studies of diagnostic test accuracy to be done, comparing DTI to other available tests such as electrophysiology, ultrasound and more.

It is widely known that diffusion metrics in the brain are strongly dependent on preprocessing pipelines (i.e., software)^79^ but still there is no consensus on the minimum or indeed ideal suite of corrections to perform. This issue is compounded in the limb owing to an absence of research on the topic and hardware limitations. The majority of the included studies did not describe any form of distortion correction, how the diffusion data were reconstructed or how metrics were extracted from the median nerve. Before DTI can be used clinically, variations in these pipelines should be tested and a universal pipeline and standards for reporting diffusion data should be agreed by consensus.

Some readers will criticise our choice to pool estimates of FA and MD in the presence of high statistical heterogeneity. This was done because forest plots provide an important graphical representation of the variability of measurements in relation to experimental conditions (e.g., b-values and N_D_), they summarise a large amount of information in an easy-to-interpret format and moreover, meta-regression facilitates the exploration of heterogeneity.

## Conclusions

We provide summary estimates of the normal FA and MD of the median nerve in asymptomatic adults. Furthermore, we show that diffusion throughout the length of the median nerve becomes more isotropic in patients with CTS, with the largest differences at the midpoint of the carpal tunnel at the level of the pisiform bone.

## Supplementary Information

Supplementary Information.

## Data Availability

The raw data are available via the Open Science Framework (https://osf.io/vqwkp/). The statistical syntax is available from the senior author (RGW) upon request.
