# Supplementary material for: Meta-analysis of the normal diffusion tensor imaging values of the median nerve and how they change in carpal tunnel syndrome
Source: Sci Rep. 2021 Oct 22;11:20935. doi: 10.1038/s41598-021-00353-z (PMC8536657; doi:10.1038/s41598-021-00353-z)
Supplement: Supplementary file 1 — Supplementary Information. [file 41598_2021_353_MOESM1_ESM.docx]

**eTable 1.** Characteristics of the included studies

| **Study** | **Country** | **Participants, n (M:F, mean age [SD])** | **Scanner brand & model (Tesla)** | **Image acquisition** | **Data processing & reconstruction methods** | **Region of Interest location (size)** | **Raters (years of experience)** |
| --- | --- | --- | --- | --- | --- | --- | --- |
| Andreisik  2008 | Canada | Healthy n=15  (7:8, 31.21 [6]) | GE Signa Excite HD (1.5T) | Single-shot EPI, TE 103ms, TR 7000ms, Slice thickness 4mm, In-plane resolution 1.875x1.875mm, Partial Fourier not described, Parallel imaging not described, signal average 2, multiple b-values (325, 500, 675, 850, 1025, 1200, 1375, 1550mm^2^/s), directions 25 | DTI Studio (release 2.4) was used but no further details given | Freehand ROI, location & size unspecified | 1 reader  (7) |
| Barcelo  2013 | France | Healthy n=20 (8:12, 33.7 [unspecified])  Patients n=15 (3:12, 50.8  [unspecified]) | Philips Achieva (3T) | Single-shot EPI, TE 55ms, TR 5855ms, slice thickness 1.8mm, in-plane resolution 0.67mmx0.76mm, partial Fourier not described, parallel imaging not described, signal averaging not specified, b-value 1000mm^2^/s, directions 15 | DTI data were processed with Philips PRIDE but no further details were given | DRUJ, Pisiform, hook of hamate (unspecified) | 2 radiologists (5 & 15) |
| Breckwoldt  2015 | Germany | Healthy n=30  (16:14, 43.5 [18.1]) | Siemens Trio and Verio  (3T) | Single-shot EPI, TE 94ms, TR 3800ms, Slice thickness 4mm, In-plane resolution 1.14x1.14mm, partial Fourier 7/8, parallel imaging not described, signal averages 2, b-value 1200mm^2^/s, directions 19 | Postprocessing was performed using FSL diffusion toolbox of the FMRIB free software library (FSL), version 5.0.6. | Unspecified (unspecified) | Unspecified (unspecified) |
| Brienza  2014 | Italy | Patients n=15 (4:11, 37.5  [3.43]) | Siemens Verio (3T) | Single-shot EPI, TE 87ms, TR 7600ms, slice thickness 5mm, in-plane resolution 1.81mmx1.81mm, partial Fourier not described, parallel imaging not described, signal averages 4, b-value 1000mm^2^/s, directions 6 | Tractography used the Runge-Kutta fiber assignment by continuous tracking (FACT) algorithm (Neuro 3D, Siemens) | Pisiform (unspecified) | 2 senior radiologists |
| Bulut 2014 | Turkey | Healthy n=48  (11:37, 41.85 [7.81])  Patients n=72  (19:53, 43.07 [7.4]) | Philips Achieva (1.5T) | Single-shot EPI, TE 21ms, TR 10254ms, slice thickness 4mm, in-plane resolution 0.45x0.45mm, Partial Fourier not described, Parallel imaging not described signal averages 3, b-value 1000mm^2^/s, directions 32 | Realignment & co-registration for reduction of motion artefacts and distortion. Fibertrack utilised. (Extended MRWorkSpace, version 2.5.3.0.) | Manual placement (unspecified) | 2 radiologists (5 & 11) |
| Cingoz 2018 | Turkey | Healthy n=9  (unspecified, 54 [15.74])  Patients n=31  (unspecified, 50 [12.43]) | Siemens Magnetom Avanto (1.5T) | Single-shot EPI, TE 84ms, TR 5400ms, slice thickness 3mm, in-plane resolution 1.43x1.56mm, Partial Fourier not described, parallel imaging not described, signal averages 3, b-value 800mm^2^/s, directions 20 | A deterministic fiber tracking algorithm was used (DSI studio). | DRUJ, Pisiform,  Hook of Hamate  (unspecified) | Not described |
| Ding 2015 | China | Healthy n= 10  (5:5, unspecified) | GE, model not specified (3T) | Single-shot EPI, TE 92ms, TR 8000ms, slice thickness 2.6mm, in-plane resolution 0.47x0.47mm, partial Fourier not described, SENSE parallel imaging with acceleration factor 2, signal averages 4, b-value 1000mm^2^/s, directions 6 and 25 | Not described | DRUJ, Pisiform, Hook of Hamate  (unspecified) | 2 radiologists (>20 & >20) |
| Edward  2020 | Egypt | Healthy n=15  (10:5, 45.67 [7.77]) | Phillips Achieve Sense (1.5T) | Single-shot EPI, TE not described TR not described, slice thickness not described, In-plane resolution not described, partial Fourier not described, parallel imaging not described, signal average not described, b-value 2000mm^2^/s, directions 32 | Not described | DRUJ & Hook of Hamate (unspecified) | 1 radiologist  (unspecified) |
| Filli  2015 | Switzerland | Healthy n=8  (6:2, 29.4 [unspecified) | Siemens Magnetom Skyra (3T) | Single-shot EPI, TE 74ms, TR 5200ms, slice thickness 3mm, in-plane resolution 1.4x1.4mm, partial Fourier 5/8, parallel imaging with CAIPIRINHA, signal averages 5, b-value 1000mm^2^/s, directions 20 | Not described | DRUJ, Pisiform, Hook of Hamate  (not described) | 2 readers  (unspecified) |
| Guggenberger  2012 | Switzerland | Healthy n=45  (15:30, 41.13 [8.4])  Patients n=15  (5:10, 52.1 [15.24]) | Phillips Achieva X series (3T) | Single-shot EPI, TE 40ms, TR 10123ms, slice thickness 4mm, in-plane resolution 0.54x0.54mm, partial Fourier not described, SENSE parallel imaging with acceleration factor 2, signal averages 2, b-value 1200mm^2^/s, directions 15 | Not described | DRUJ, Pisiform, Hook of Hamate  (unspecified) | 2 radiologists  (2 & 2) |
| Gruggenberger  2013 | Switzerland | Healthy n=16, (7:9, 30.6  [ 5.3]) | Siemens Magnetom Verio, GE Discovery MR 750 and Philips Ingenia (3T) | Single-shot EPI, TE 91ms, TR 9200ms, slice thickness 4mm, in-plane resolution 1.2x1.2 mm, partial Fourier not described, parallel imaging not described signal averages 2, b-value 1200mm^2^/s, directions 20 | Ingenia MR systems workstation with software release 4.1.1.1. using previously described post-processing techniques | DRUJ, Pisiform, Hook of Hamate  (unspecified) | 2 radiologists  (3&5) |
| Heckel  2015 | Germany | Healthy n=30 (13:17, 39.3 [15.5]) | Siemens Magnetom Trio (3T) | Single-shot EPI, TE 99ms, TR 3800ms, slice thickness 4mm, in-plane resolution 1.17mm x1.17mm, partial Fourier not described, parallel imaging not described signal averages 2, b-value 1200mm^2^/s, 19 directions with a monopolar waveform | DTI postprocessing and quantitative analysis was performed using FSL Diffusion Toolbox (FDT) | DRUJ, Pisiform, Hook of hamate (unspecified) | 2 readers (unspecified) |
| Hitunen  2005 | Finland | Healthy n=4  (no further details specified) | GE Signa VH (3T) | Single-shot EPI, TE 84ms, TR 5000ms, slice thickness 3.5mm, in-plane resolution 1.56mm x1.56mm, partial Fourier not described, Parallel imaging not described, signal averages 4, b-value 1000 mm^2^/s, directions 13 | No corrections described. Tractography based on a FA threshold of 0.4. Custom ROIs (unspecified) | Pisiform (unspecified) | Unspecified (unspecified) |
| Hiltunen  2012 | Finland | Healthy n=12 (3:9, 46  [unspecified])  Patients n=12 (3:9, 47  [unspecified]) | GE Signa HTXT (3T) | Single-shot EPI, TE 86ms, TR 10000ms, slice thickness 3mm, in-plane resolution 1.88mmx1.88mm, partial Fourier not described, parallel imaging not described, signal averages 3, b-value 1000mm^2^/s, directions 25 | The 3D course of the nerve was tracked with tractography computed with the FACT algorithm using DTIStudio | DRUJ, Pisiform  Hook of hamate | 1 radiologist (unspecified) |
| Kabakci  2007 | Turkey | Healthy n=20,  (unspecified, 27.5  [unspecified])  Patient n=2  (unspecified) | Philips Intera Achieva | Single-shot EPI, TE 90ms, TR 4600ms, slice thickness 4mm, in-plane resolution 1.09mmx10.9mm, partial Fourier not described, parallel imaging not described signal averages 3, b-value 1000mm^2^/s, directions 32 | No corrections described. The PRIDE (Philips Medical Systems) was used but no further details were provided | DRUJ. ROIs “larger than the nerve” (unspecified) | Unspecified (unspecified) |
| Khalil  2008 | France | Healthy n=13  (2:11, 55 [6.58])  Patient n=13  (2:11, 54 [7.77]) | Phillips Achieva (1.5T) | Single-shot EPI, TE 89ms, TR 3198ms, slice thickness 2mm, in-plane resolution 0.78x0.77mm, partial Fourier 6/8, SENSE parallel imaging with acceleration factor 2, signal average not described, b-value 400mm^2^/s, directions 32 | Eddy-current and motion artifact corrected using Automated Image Registration software (Philips Pride Diffusion Registration and IDL, ITT, Boulder, CO) | Not described  (unspecified) | 2 radiologists  (3 & 13) |
| Klauser  2017 | Austria | Healthy n=14 (4:10, 57.8 [13.2])  Patients n=40 (12:28, 58.5 [8.8]) | Siemens MAGNATOM Skyra (3T) | Redout-segment EPI (RESOLVE), TE 93ms, TR 3400ms, slice thickness 3mm, in-plane resolution 0.9mmx0.9mm, partial Fourier not described, parallel imaging not described signal averages 2, b-value 1000mm^2^/s, directions 12 | Not described | Hamate  (unspecified) | 1 radiologist (10) |
| Koh  2014 | South  Korea | Healthy n=42  (36:6, 52.8 [7.58])  Patient n=42  (11:31, 52.4 [7.58]) | Phillips Achieva  (3T) | Single shot echo planar, TE 91ms, TR 7049ms, slice thickness 2mm, in-plane resolution 1.1x1.1mm, partial Fourier not described, SENSE parallel imaging with acceleration factor 2, signal averaging not described, b-value 1000mm^2^/s, directions 15 | Realignment using diffusion registration software (Extended MRWorkSpace, version 2.5.3.0.) | DRUJ, Pisiform,  Hook of Hamate  (unspecified) | 2 radiologists  (unspecified) |
| Kwon  2015 | South Korea | Healthy n=26  (3:23, 50.3 [9.3])  Patient n=26  (3:23, 53.6 [8.9]) | Phillips Achieva  (3T) | Single-shot EPI, TE 91ms, TR 7266ms, slice thickness 2mm, in-plane resolution 1x1mm, partial Fourier not described, parallel imaging not described, signal averages 5, b-value 1000mm^2^/s, directions 15 | Reduction of motion and eddy current artifact using co-registration (Achieva release 3.2.1.1, Philips Healthcare) | Pisiform, Hook of Hamate  (unspecified) | 2 radiologists  (4 & 10) |
| Linberg  2013 | France | Healthy n=10 (no further details specified) | Not specified | Single-shot EPI, TE 100ms, TR 7000ms, slice thickness 4mm, in-plane resolution 0.4mmx0.4mm, partial Fourier not described, SENSE parallel imaging with acceleration factor 2, signal averages 7, not described b-value - 1000mm^2^/s, Directions 6, gradient waveform not described | Not described | DRUJ  Pisiform  Hook of hamate | Unspecified (unspecified) |
| Naraghi  2013 | Canada | Patients n=8 (1:7, 64  [unspecified]) | Signa Excite HD GE Healthcare  (1.5T) | Single-shot EPI, TE – 103ms, TR – 7000ms, Slice thickness – 4mm, In-plane resolution – 1.875mmx1.875mm, Partial Fourier not described, Parallel imaging not described, Signal average not described b-value – 1025mm^2^/s, Directions 25, gradient waveform not described | Not described | DRUJ  Pisiform  Hook of hamate | Unspecified (unspecified) |
| Paniandi  2018 | Malaysia | Healthy n=8  (0:8, unspecified)  Patient n=8  (0:8, unspecified) | GE Healthcare Signa HDx (3T) | Sequence unspecified, TE 30ms, TR 7650ms, slice thickness 4mm, in-plane resolution 0.55x0.55mm, partial Fourier not described, parallel imaging not described, no signal averaging, b-value 1000mm^2^/s, directions 32 | 3D SLICER software (v4.5.01) used for tracking and DTI measurements | DRUJ, Pisiform,  Hook of Hamate  (unspecified) | Unspecified (unspecified) |
| Razek  2016 | Egypt | Healthy n=15  (4:11, 32 [2.8])  Patient n=39  (9:30, 33 [3.02]) | Phillips Achieva (1.5T) | Single-shot EPI, TE 91ms, TR 7049ms, slice thickness 2mm, in-plane resolution 1x1mm, partial Fourier not described, SENSE parallel imaging with acceleration factor 2, signal averages 5, b-value 1000mm^2^/s, directions 15 | FiberTracking software package (Extended MR WorkSpace, version 2.5.3.0; Philips Medical Systems) was used for image analysis. | Hook of Hamate (unspecified) | 1 radiologist  (25) |
| Schmid  2018 | UK | Healthy n=6  (3:3, 30.3 [7.11])  Patient n=8  (4:4, 55.3 [11.5]) | Siemens Magnetom  (7T) | Single-shot EPI, TE 60ms, TR 3300ms, slice thickness 1.5mm, in-plane resolution 1.5x1.5mm, partial Fourier not described, GRAPPA parallel imaging with acceleration factor 2, no signal averaging, b-value 1000mm^2^/s, directions 12 | FMRIB’s TOPUP and eddy were used to correct for eddy-current-induced distortions and rigid-body movement. Corrected data underwent affine registration to morphological (SSFP) scans | DRUJ, Pisiform and Hook of Hamate. ROI not further described | 2 investigators  (1 & 7) |
| Stein  2009 | Israel | Healthy n=17  (7:10, 26 [4])  Patient n=9  (3:6, 44 [13]) | GE, model not specified (3T) | Single-shot EPI, TE 81ms, TR 10000ms, slice thickness 3mm, in-plane resolution 1.09x1.09mm, partial Fourier not described, parallel imaging not described, signal averages 10, b-value 1000mm^2^/s, directions 6 | Not described | DRUJ, Pisiform  Hook of Hamate  (unspecified) | Unspecified (unspecified) |
| Tasdelen  2011 | Turkey | Healthy n=26 (9:17, 34 [9])  Patient n=38  (4:34, 48 [10]) | Phillips Achieva Interna  (3T) | Single-shot EPI, TE 90ms, TR 4600ms, slice thickness 4mm, in-plane resolution 1.09x1.09mm, partial Fourier not described, parallel imaging not described, signal average 3, b-value 1000mm^2^/s, directions 16 | PRIDE (Philips Medical Systems) was used but no further details were provided | DRUJ (circular ROI, no further details) | Radiologist and radiology technician (not described). |
| Wang  2012 | Taiwan | Healthy n=19  (7:12, 52.3 [8.4])  Patient n=21  (5:16, 55.5 [9]) | Phillips Achieva (1.5T) | Single-shot EPI, TE 77ms, TR 3239ms, slice thickness 2mm, in-plane resolution 0.57x0.57mm, partial Fourier 5/8, parallel imaging not described, signal averages 2, b-value 400mm^2^/s, directions 32 | ‘Realignment’ using the manufacturer’s registration software (Extended MR WorkSpace, version 2.5.3.0) | DRUJ, Pisiform, Hook of Hamate (unspecified) | 2 radiologists  (8 & 14) |
| Wafaie  2018 | Egypt | Healthy n=15  (7:8, 44 [14.31])  Patient n=31  (5:26, 46 [13.65]) | Philips, model not specified (1.5T) | Single-shot EPI, TE 90ms, TR 4600ms, slice thickness 4mm, in-plane resolution 1.09x1.09mm, partial Fourier not described, parallel imaging not described, Signal average 3, b-value 1000mm^2^/s, directions 32, gradient waveform not described | Not described | DRUJ, Pisiform, Hook of Hamate  (unspecified) | 2 radiologists  (7 & 12) |
| Yao  2009 | USA | Healthy n=13 (unspecified, 37 [unspecified]) | Philips Achieva (3T) | Readout-segment EPI, TE 83ms, TR 1470ms, slice thickness 3mm, in-plane resolution 1mmx0.7mm, partial Fourier not described, SENSE parallel imaging was used but the acceleration factor was not described, signal averages 3, b-value - 1000mm^2^/s, Directions 32, gradient waveform not described | Not described | DRUJ  Pisiform | An “experienced” radiologist |
| Yildrim  2014 | Turkey | Patients n=41 (11:30, 41.8 [7.0]) | Philips Achieva (1.5T) | Single-shot EPI, TE 21ms, TR 10254ms, slice thickness 4mm, in-plane resolution 0.45mmx0.45mm, partial Fourier 4/8, parallel imaging not described, 2 signal averages, b-value 1000mm^2^/s, directions 32 | Not described | Unspecified | 1 radiologist (11) |
| Zhou  2012 | USA | Healthy n=10 (5:5, 26  [unspecified]) | Philips medical systems (3T) | Single-shot EPI, TE 65ms, TR 7200ms, slice thickness 3mm, in-plane resolution 1.8mmx1.8mm, partial Fourier 6/8, SENSE parallel imaging with acceleration factor 2, Signal averages 3, b-value 1000mm^2^/s, directions 21 | Registration to reference image used to correct for eddy- current artefact (DTI package, Phillips) | Unspecified | Unspecified (unspecified) |

**eFigure 1.** Network plot showing forward citation chasing (all records citing one or more of the included articles, red) and backward citation chasing (all records referenced in one or more of the included articles, blue) generated using CitationChaser (https://estech.shinyapps.io/citationchaser/)


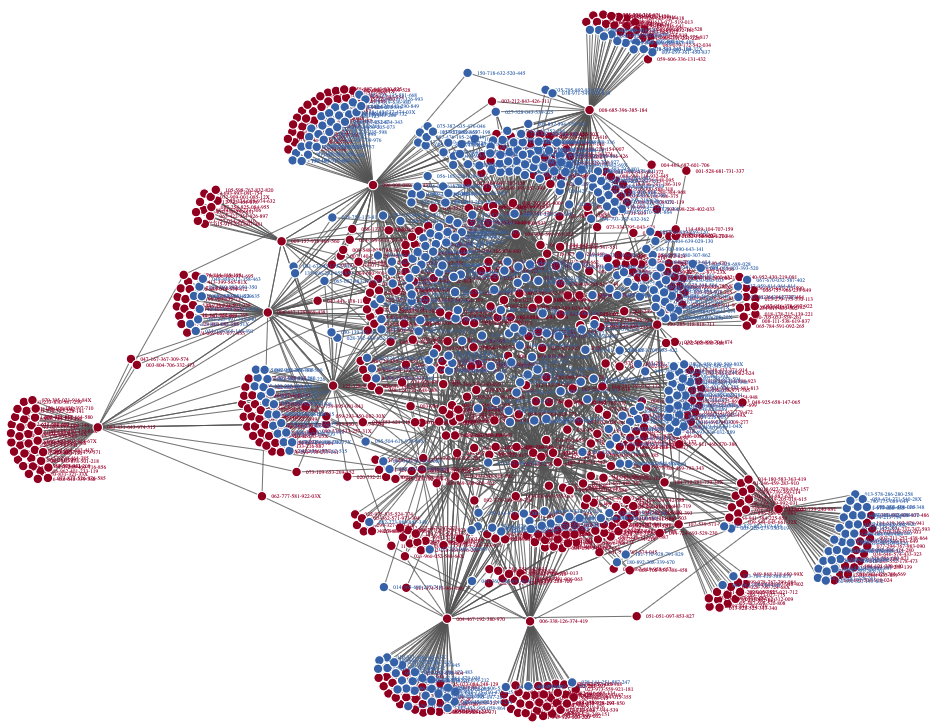


**eFIgure 2.** PRISMA 2020 flow diagram


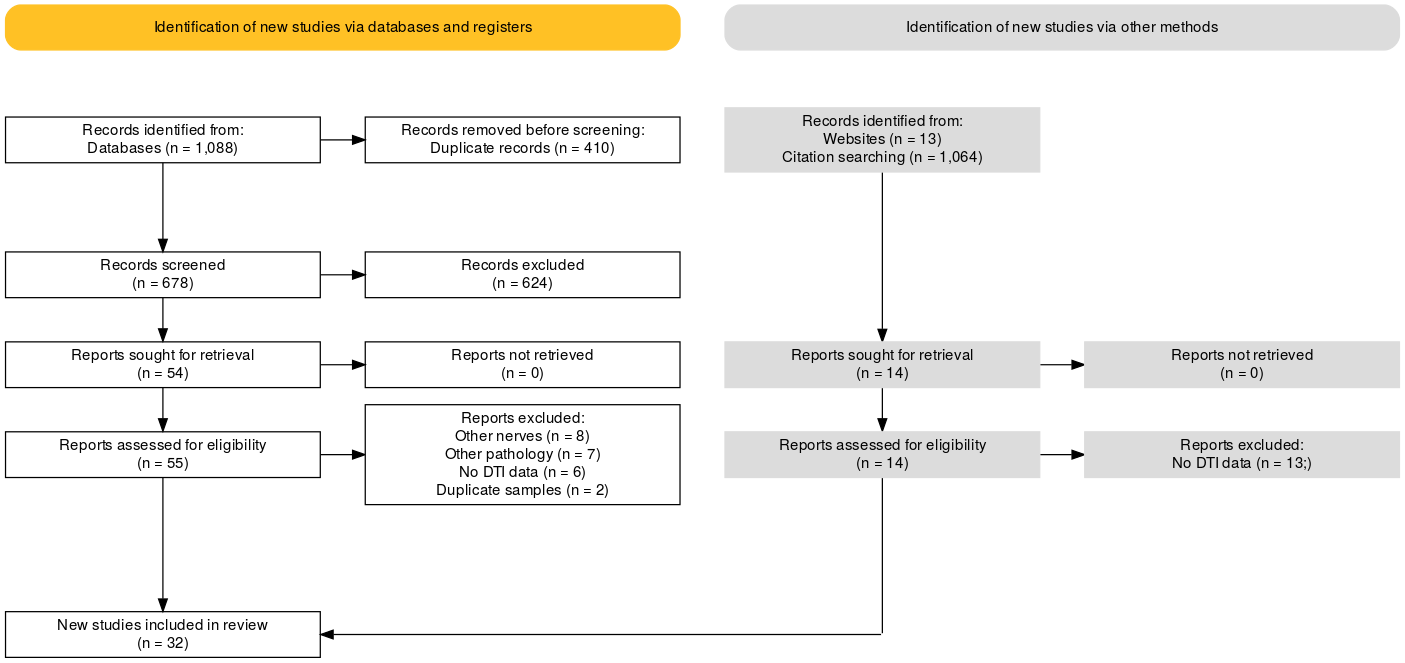


**eFigure 3.** A systematic map showing the location of the 1^st^ author of included studies, coloured by the field strength used. The map suggests a bias favouring studies in North America, Europe and Asia. This might mean that a) there was no median nerve diffusion MRI research ongoing in places such as South America, Russia and the South Pacific, b) the databases we searched failed to capture articles published in journals native to these areas or c) they were in languages which precluded them being indexed.


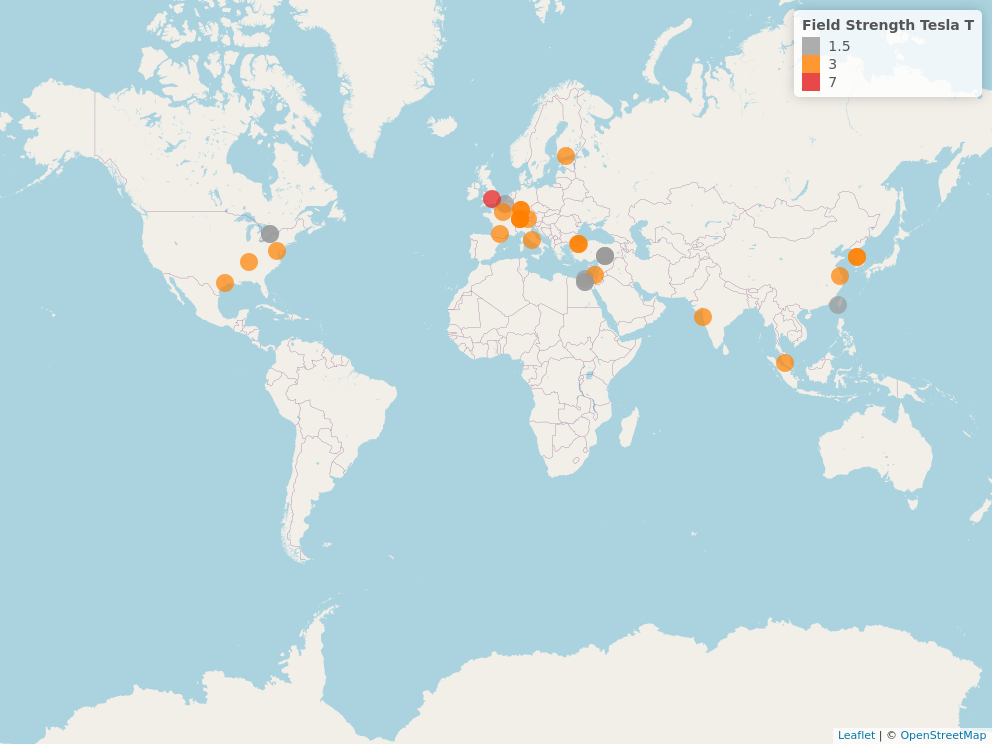


**eFigure 4.** The risk of bias within studies using the ROBINS-I tool


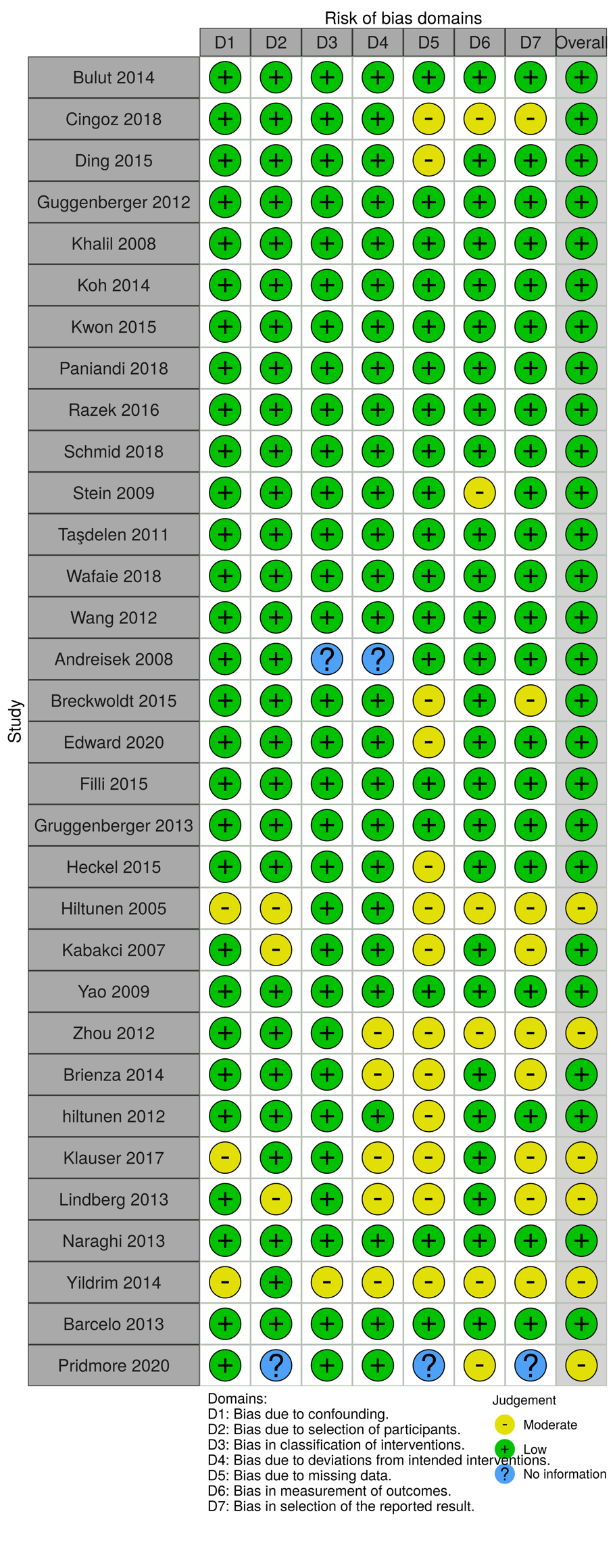


**eFigure 5.** A scatterplot of study-level estimates of fractional anisotropy against the number of diffusion-sensitising gradient directions. The size of the points corresponds to the precision (inverse variance) of the study.


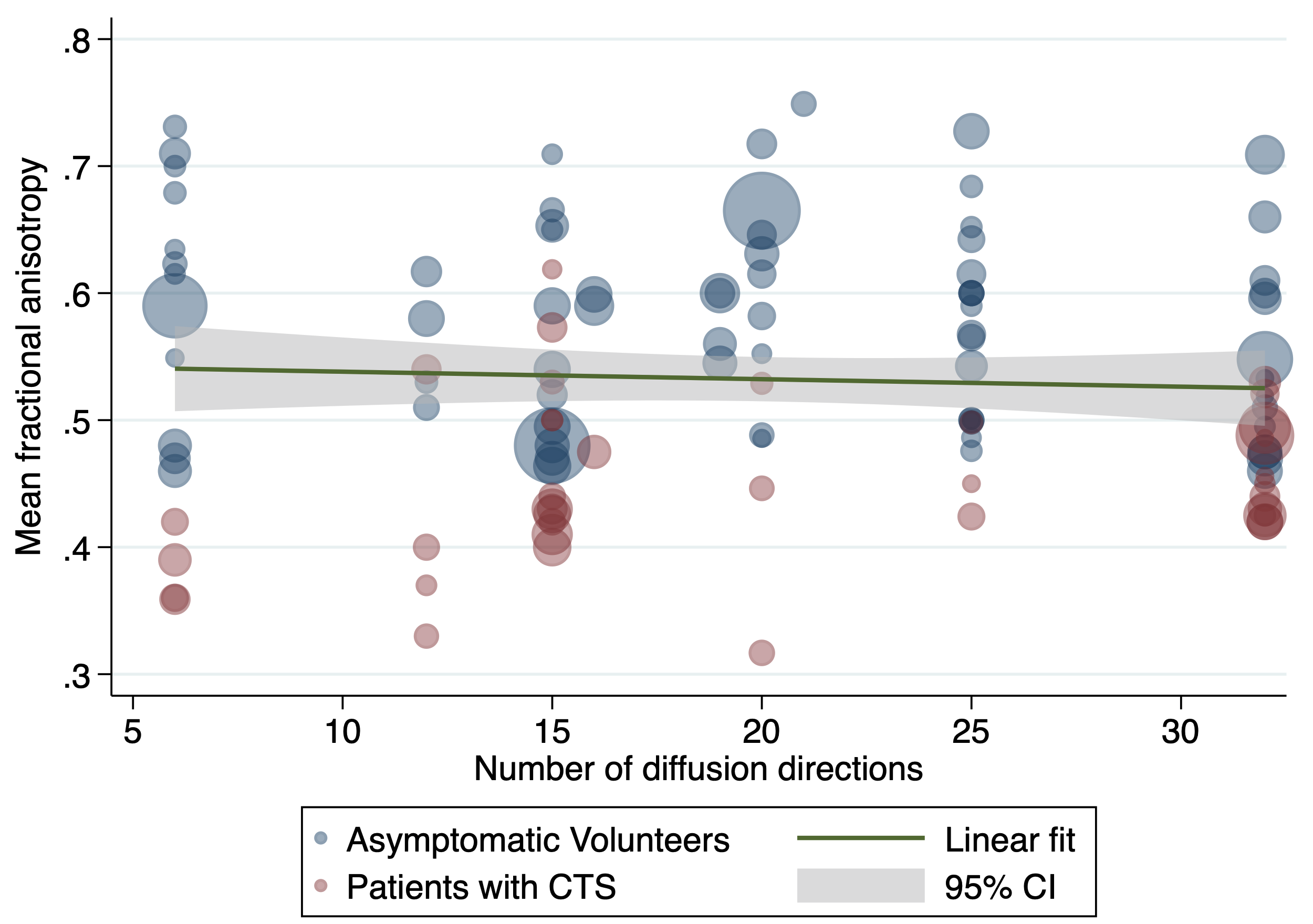


**eFigure 6.** A scatterplot of study-level estimates of mean diffusivity against the number of diffusion-sensitising gradient directions. The size of the points corresponds to the precision (inverse variance) of the study.


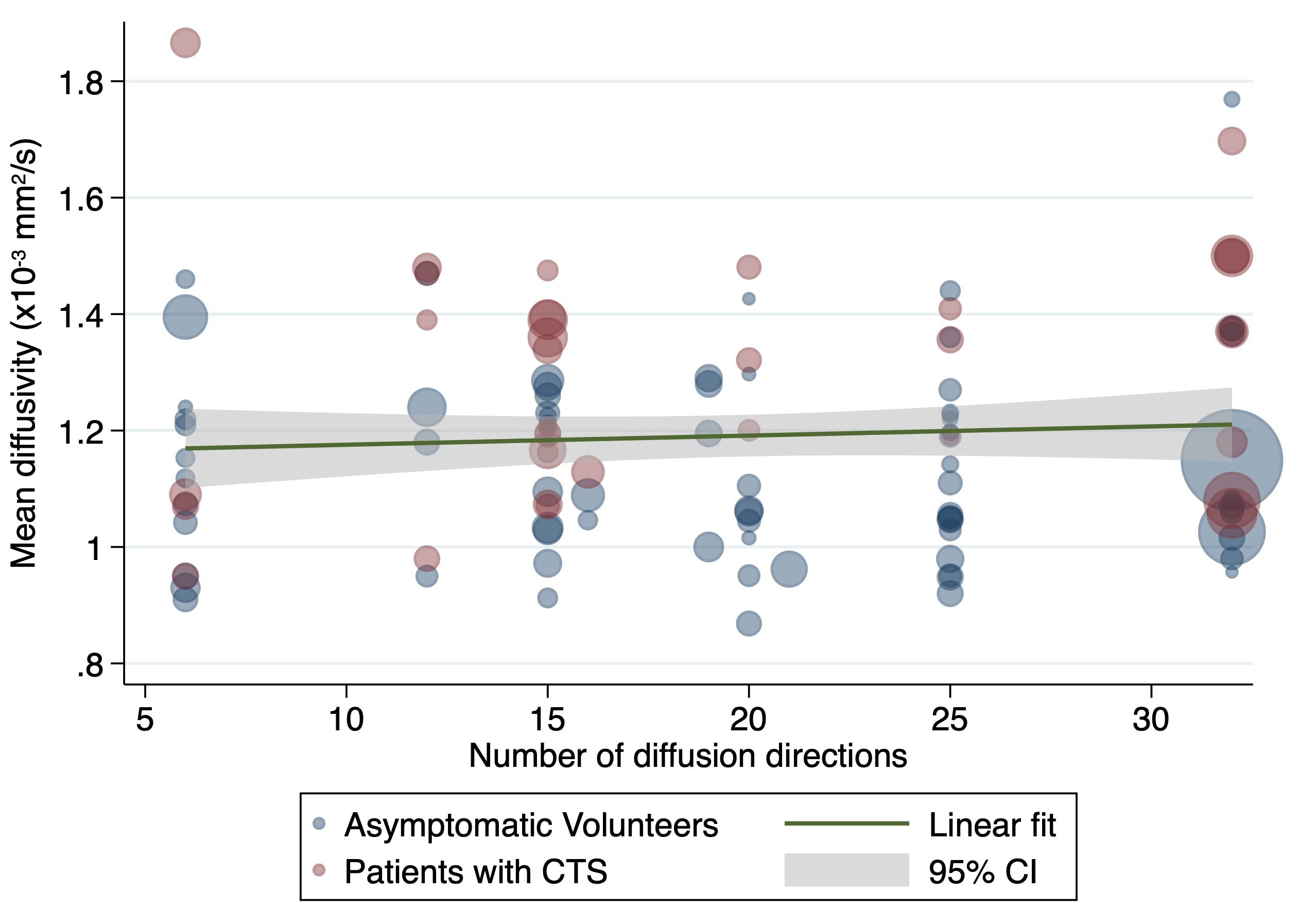


**eFigure 7.** A scatterplot of study-level estimates of fractional anisotropy against the b-value. The size of the points corresponds to the precision (inverse variance) of the study.


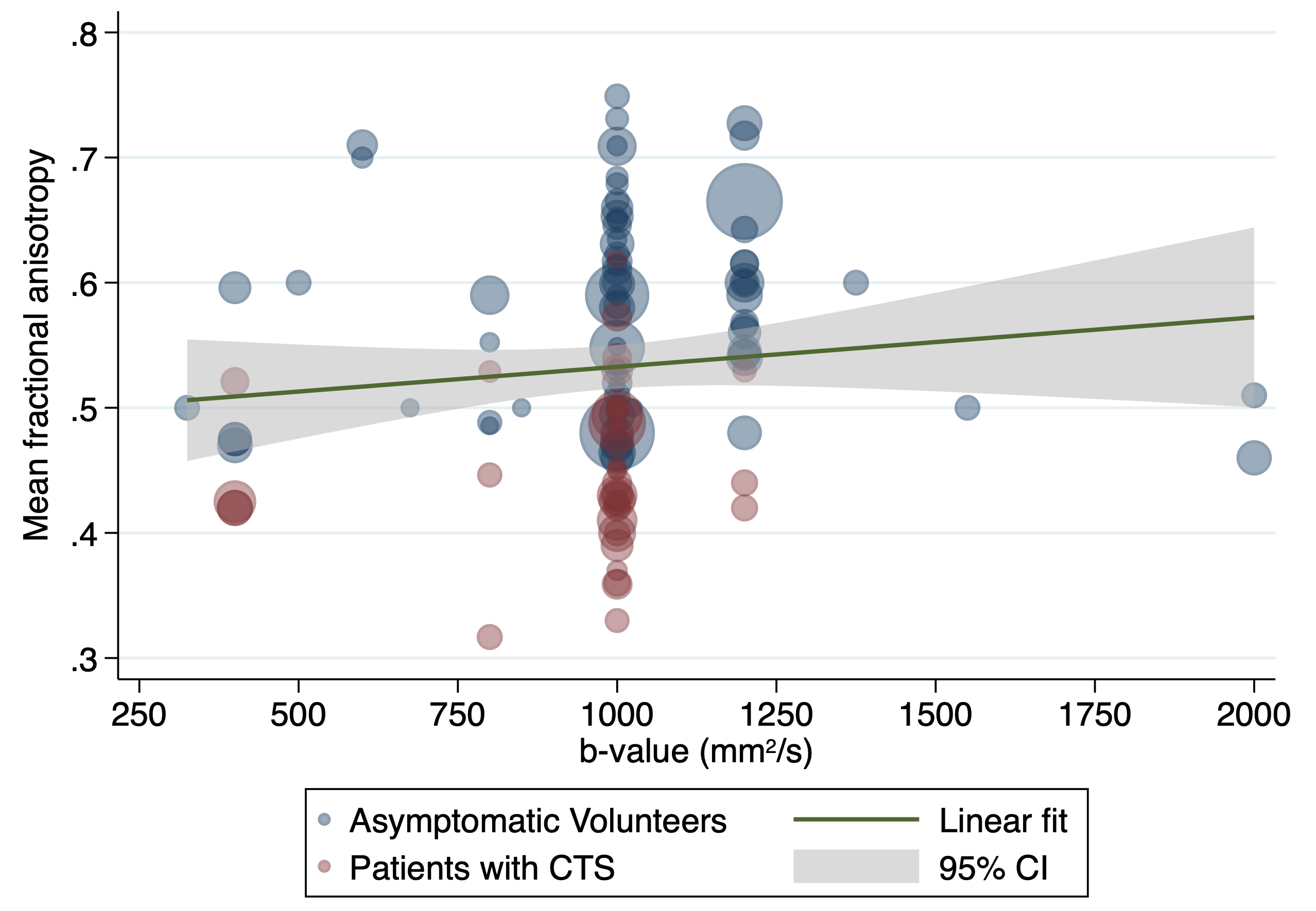


**eFigure 8.** A scatterplot of study-level estimates of mean diffusivity against the b-value. The size of the points corresponds to the precision (inverse variance) of the study.


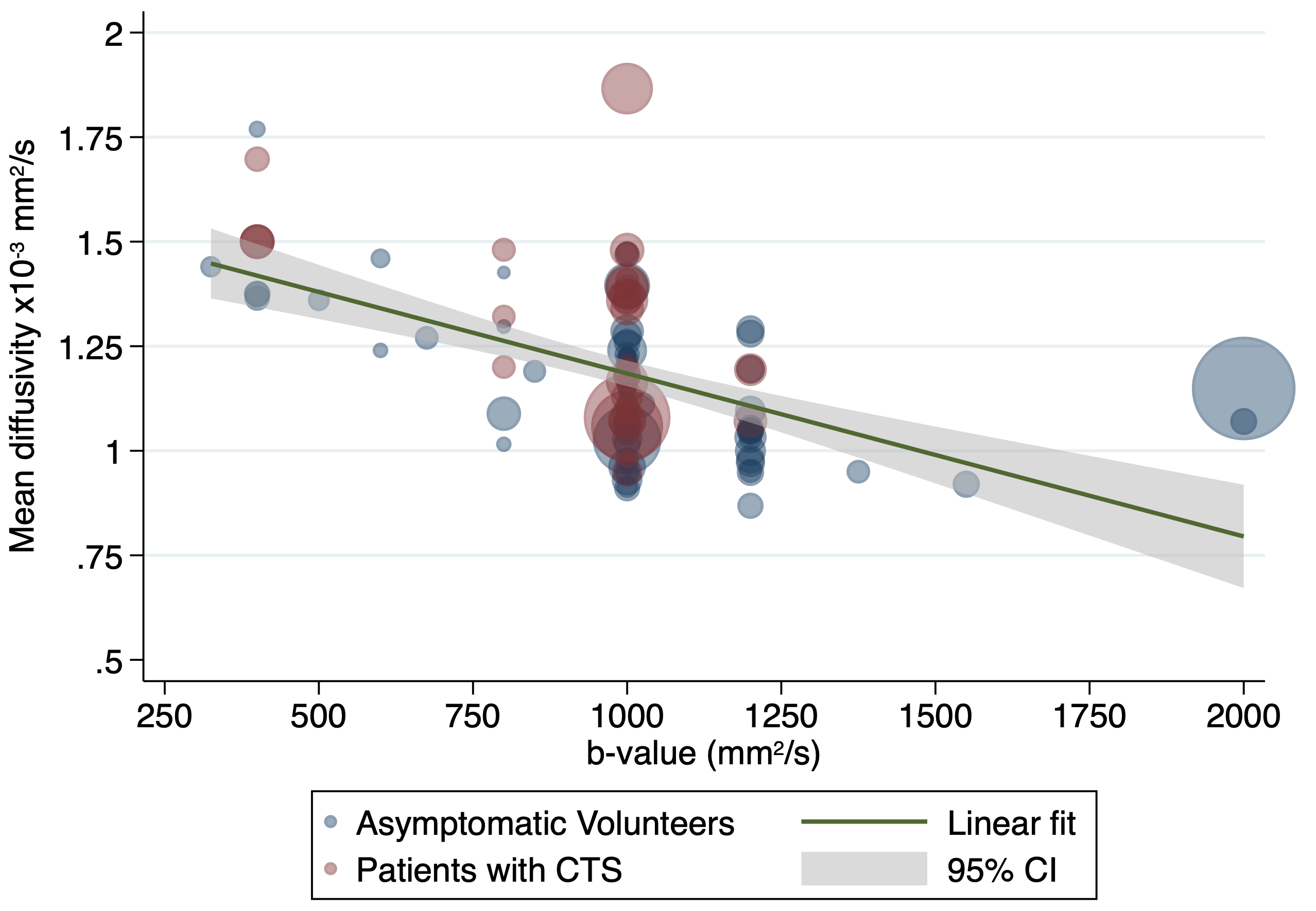


**eFigure 9.** A scatterplot of study-level estimates of fractional anisotropy against the in-plane resolution. The size of the points corresponds to the precision (inverse variance) of the study.


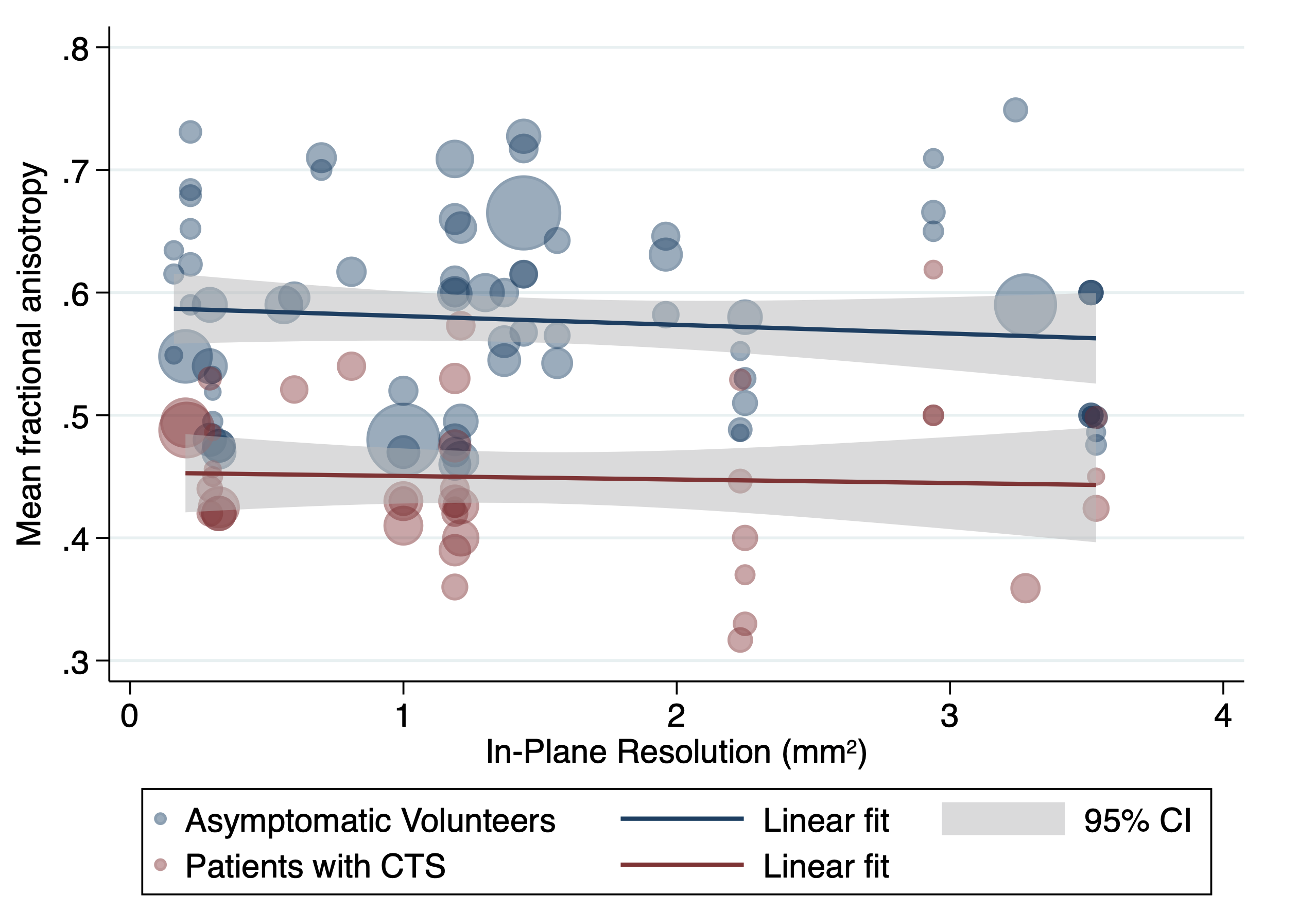


**eFigure 10.** A scatterplot of study-level estimates of mean diffusivity against the in-plane resolution. The size of the points corresponds to the precision (inverse variance) of the study.


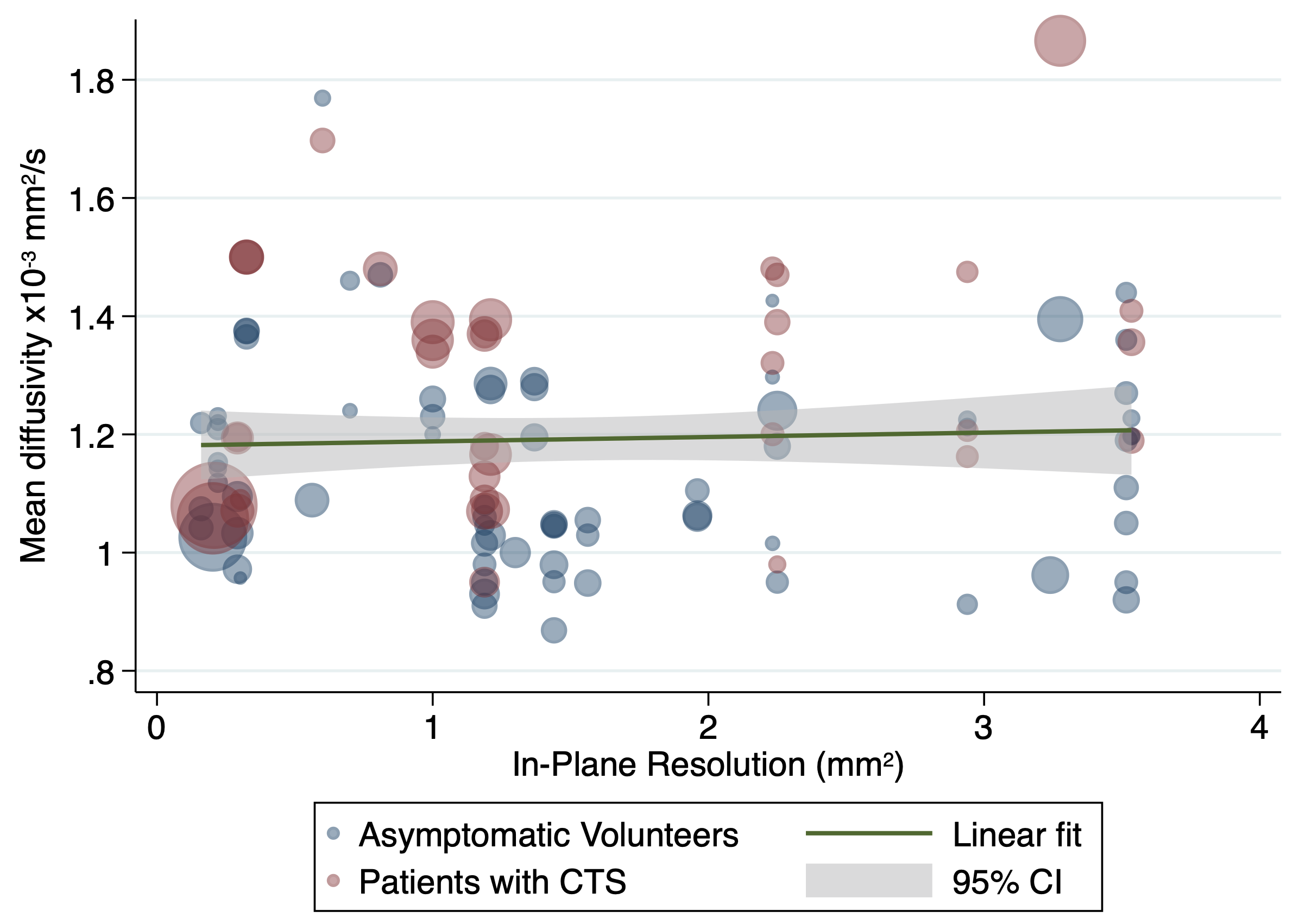


**eFigure 11.** A scatterplot of study-level estimates of fractional anisotropy against the slice thickness. The size of the points corresponds to the precision (inverse variance) of the study.


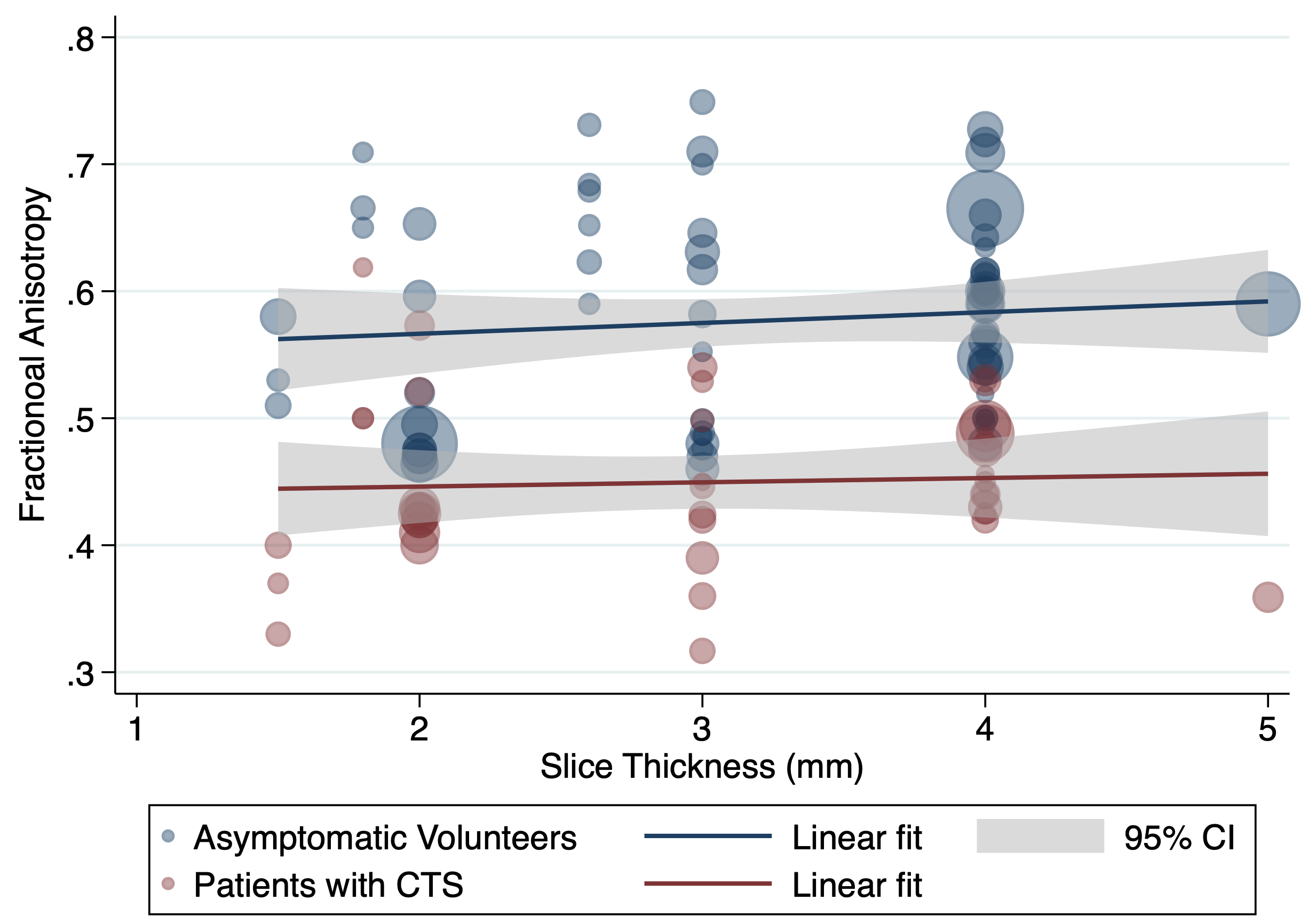


**eFigure 12.** A scatterplot of study-level estimates of mean diffusivity against the slice thickness. The size of the points corresponds to the precision (inverse variance) of the study.


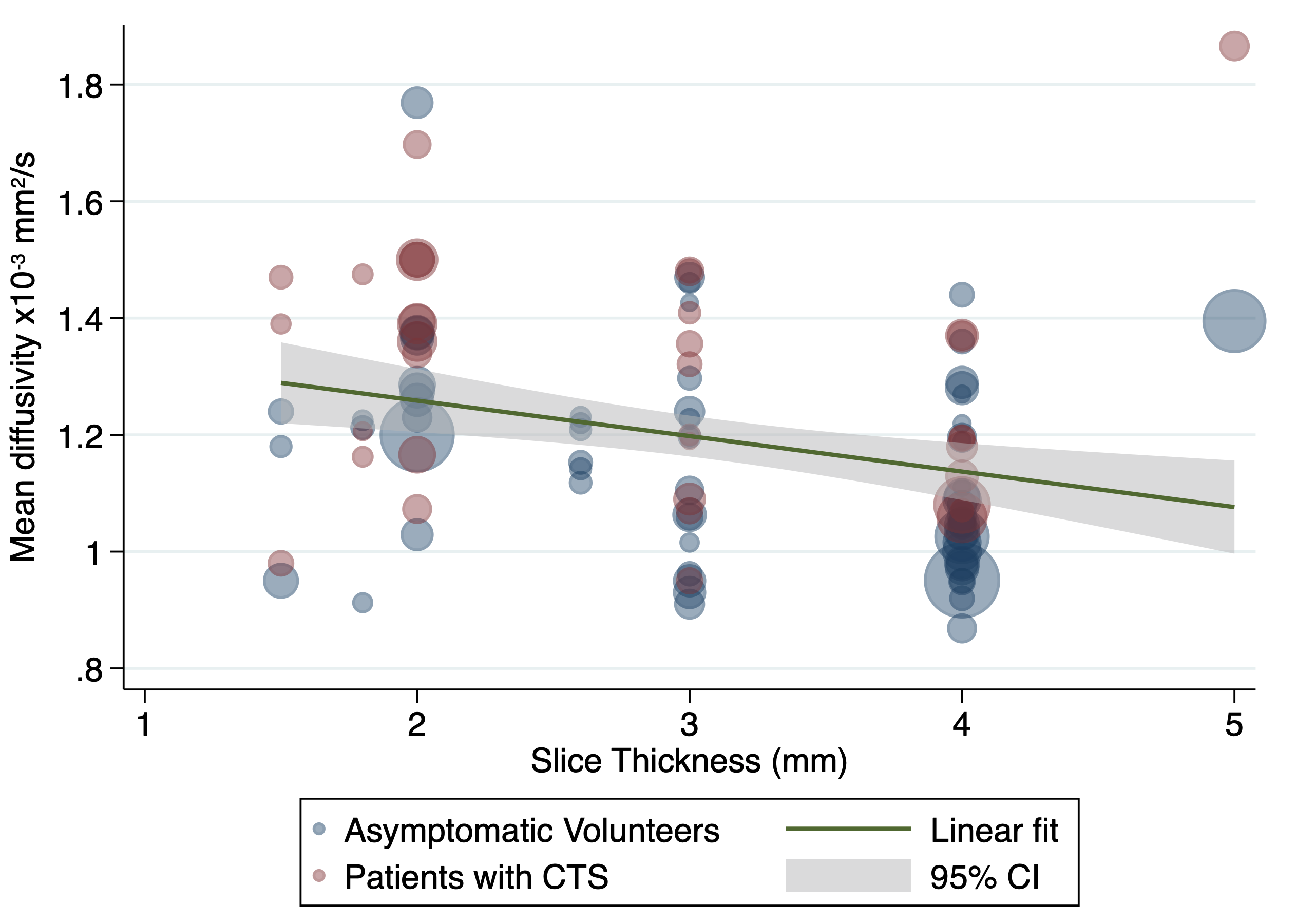


**eFigure 13.** A scatterplot of study-level estimates of fractional anisotropy against echo time. The size of the points corresponds to the precision (inverse variance) of the study.


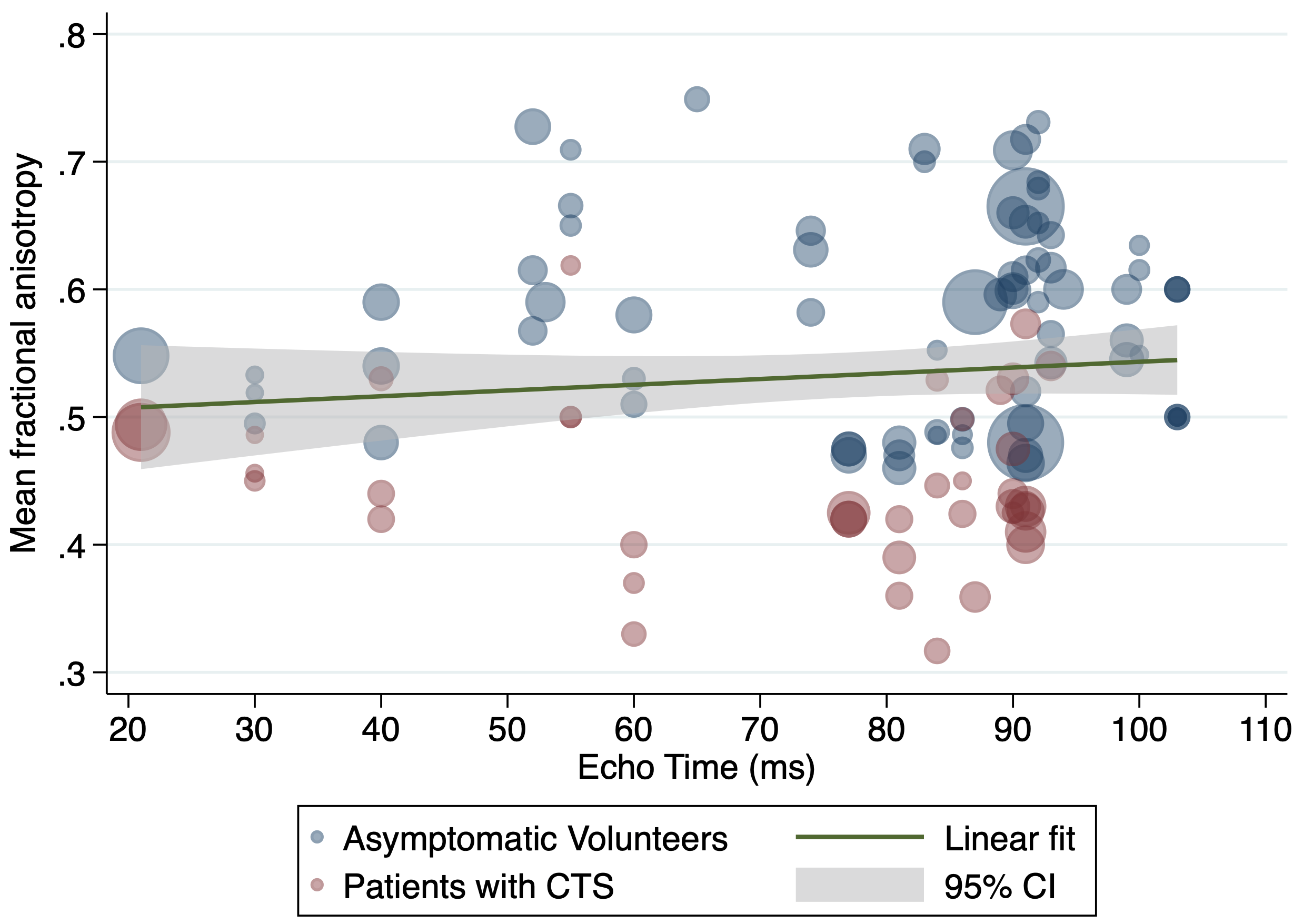


**eFigure 14.** A scatterplot of study-level estimates of mean diffusivity against the echo time. The size of the points corresponds to the precision (inverse variance) of the study.


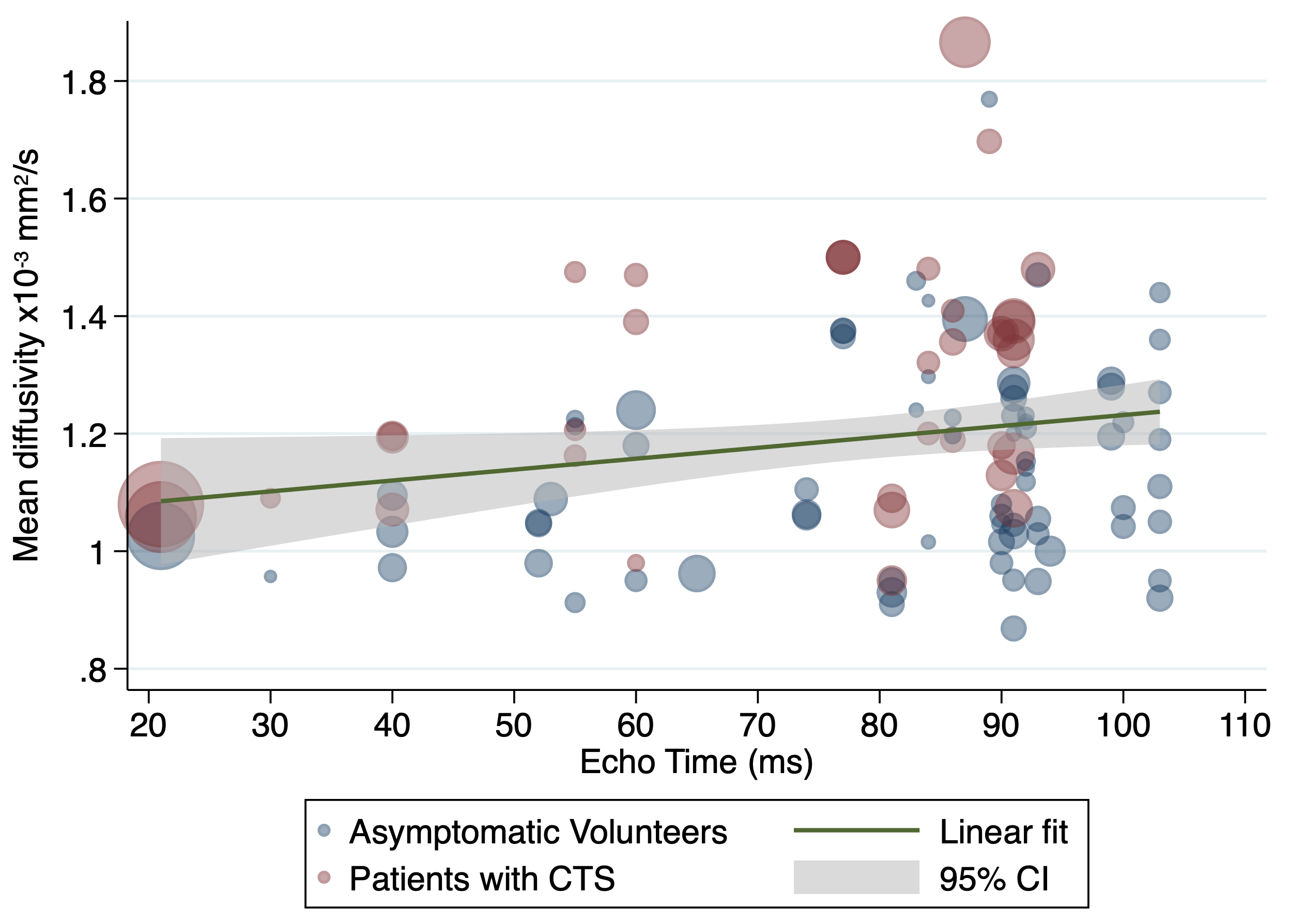


**eFigure 15.** A scatterplot of study-level estimates of fractional anisotropy against the repetition time. The size of the points corresponds to the precision (inverse variance) of the study.


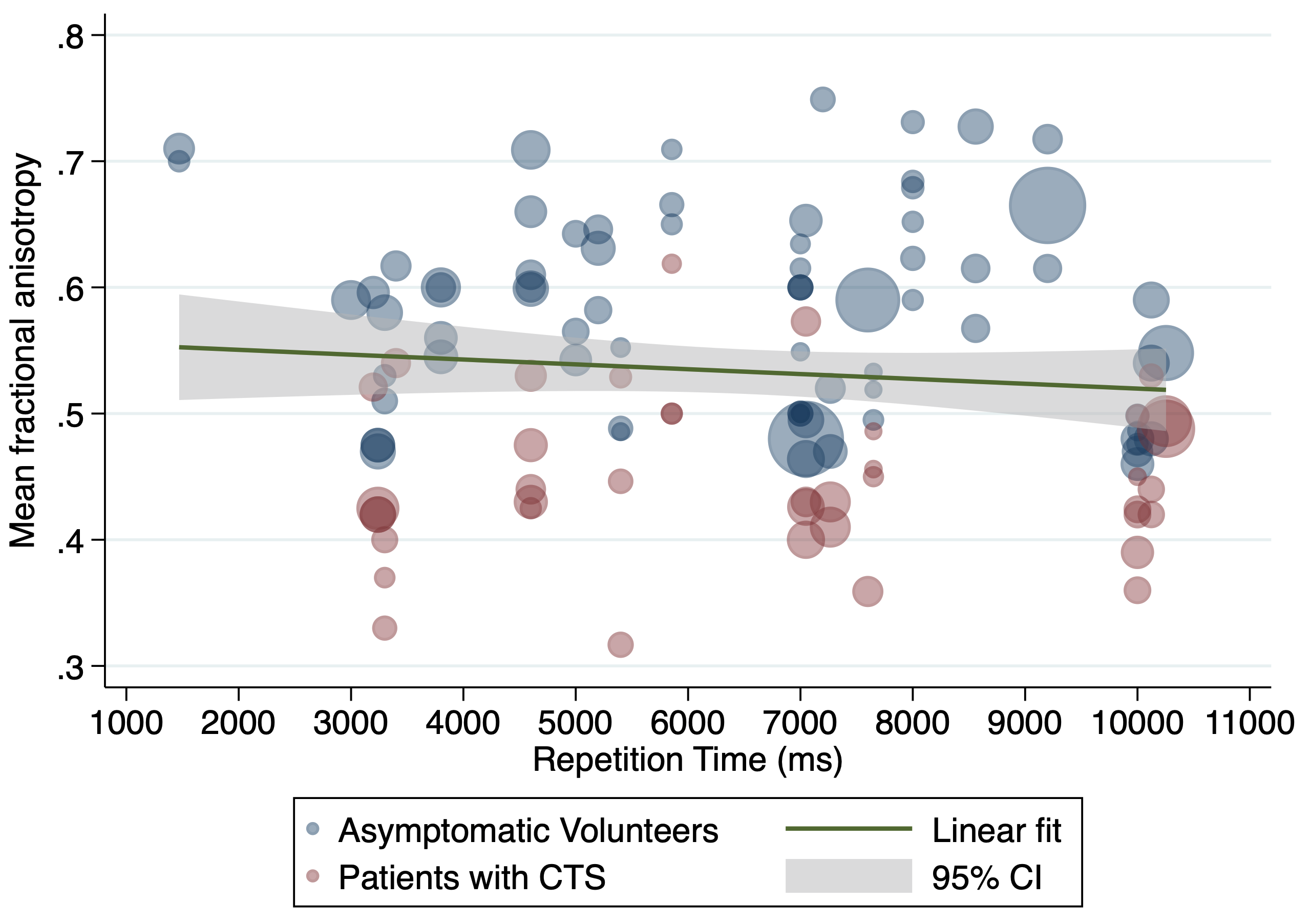


**eFigue 16.** A scatterplot of study-level estimates of mean diffusivity against the repetition time. The size of the points corresponds to the precision (inverse variance) of the study.


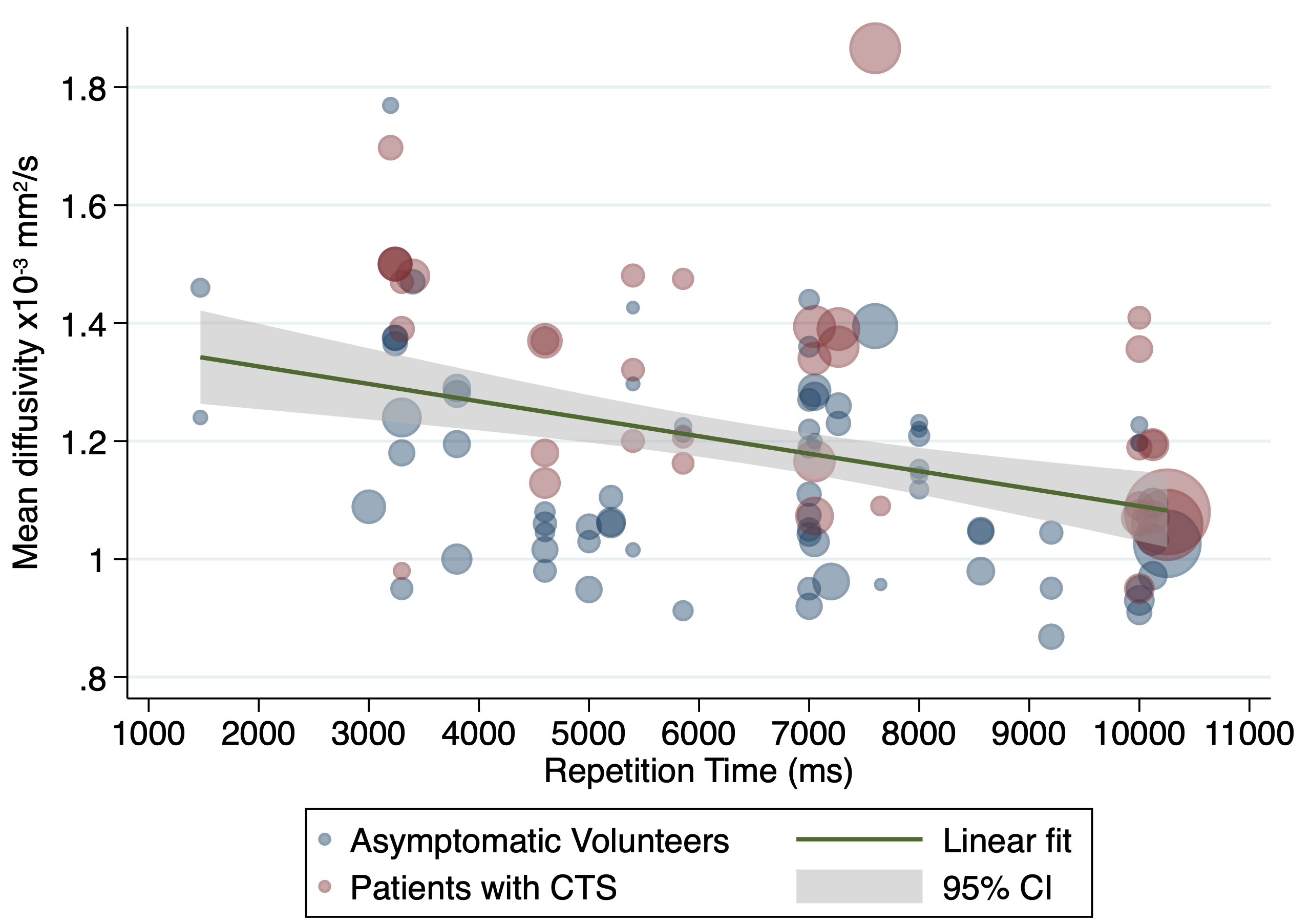


**eFigure 17.** A forest plot showing how FA changes in relation to different methods of parallel imaging

**eFigure 18.** A scatterplot of study-level estimates of fractional anisotropy against the number of signal averages. The size of the points corresponds to the precision (inverse variance) of the study.


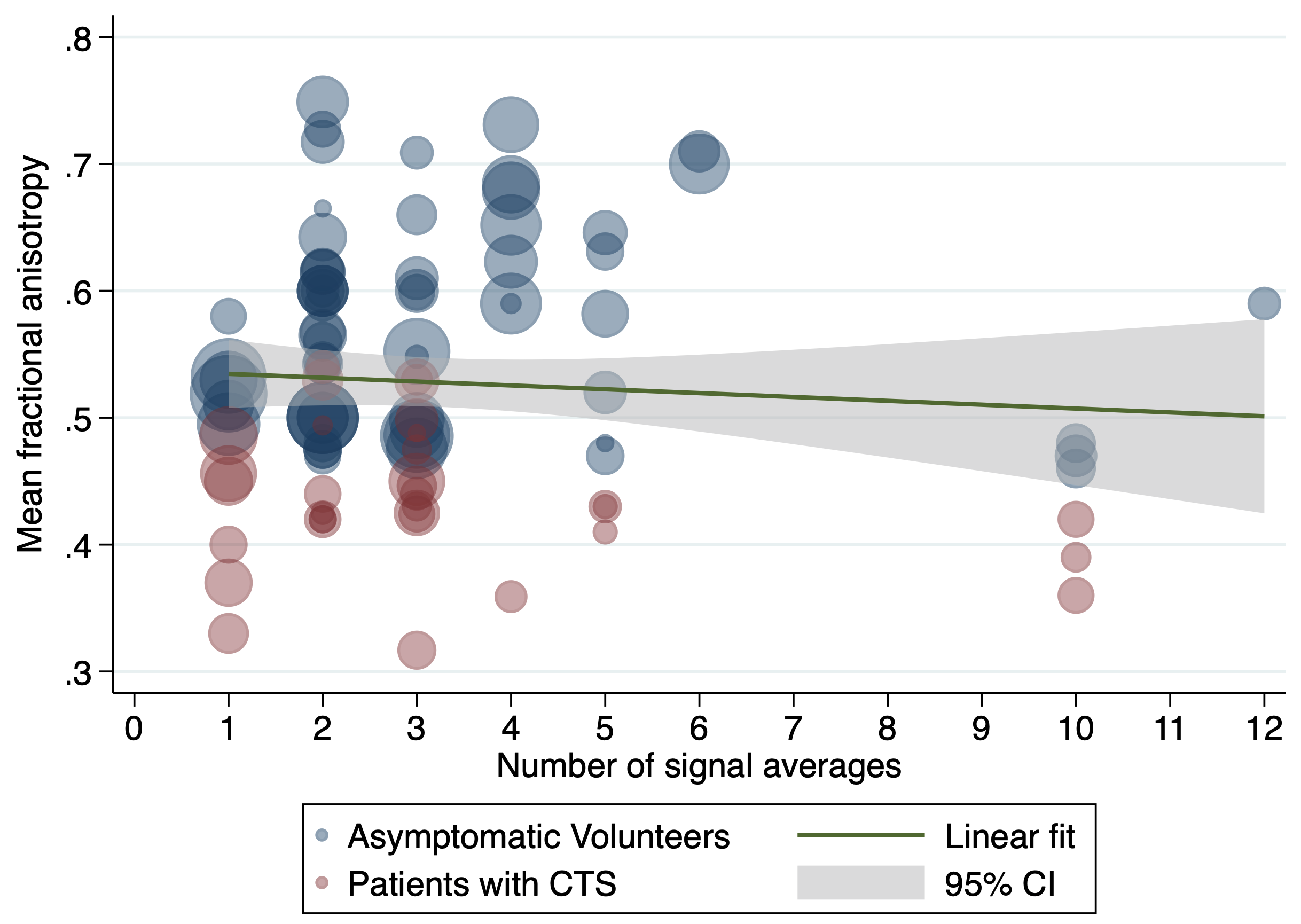


**eFigure 19.** A scatterplot of study-level estimates of mean diffusivity against the number of signal averages. The size of the points corresponds to the precision (inverse variance) of the study.


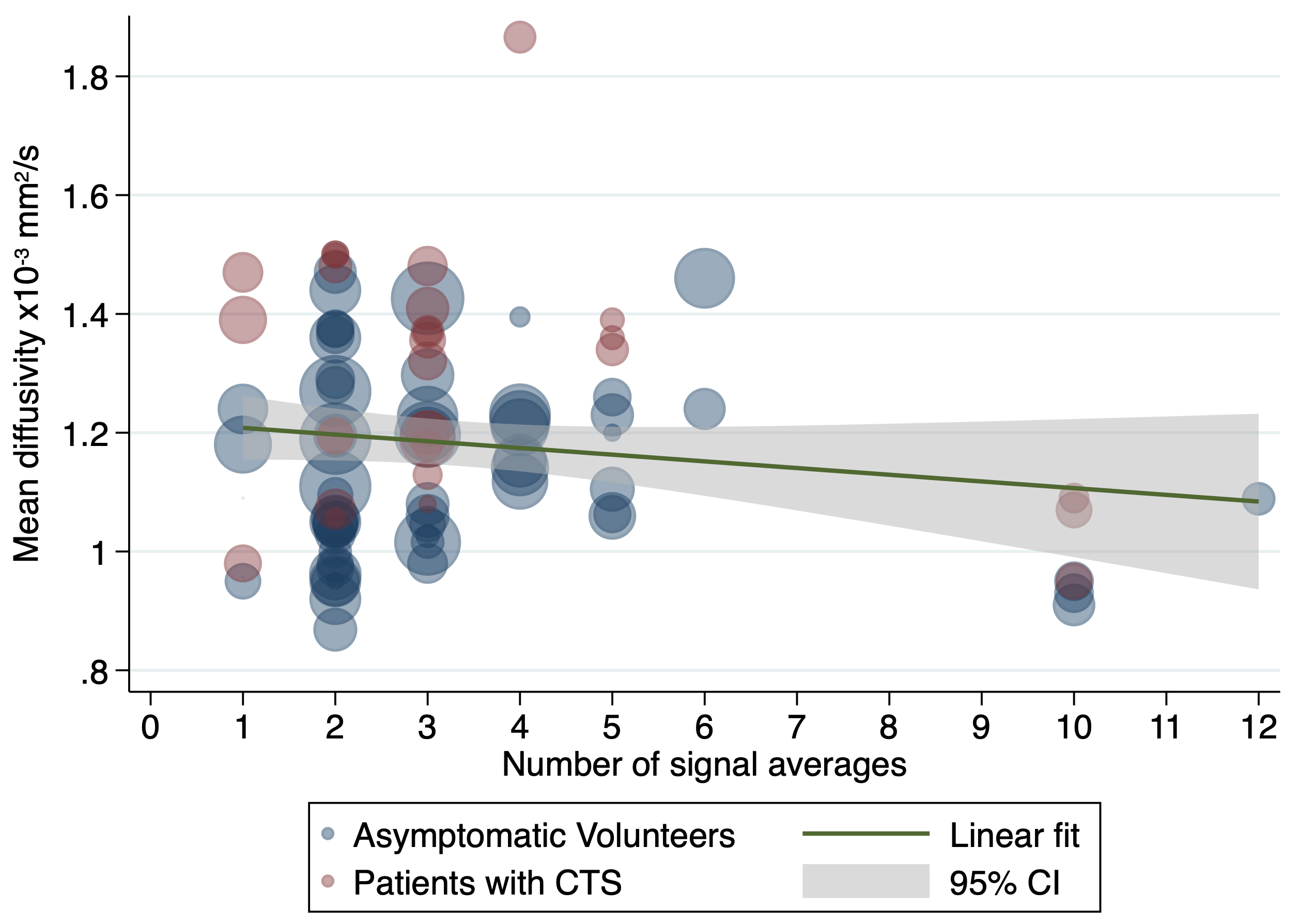


**eFigure 20.** Funnel plot with pseudo confidence intervals contoured by tau^2^ with the points coloured by the field strength (1.5 tesla red, 3 and 7 tesla blue) and scaled by the b-value


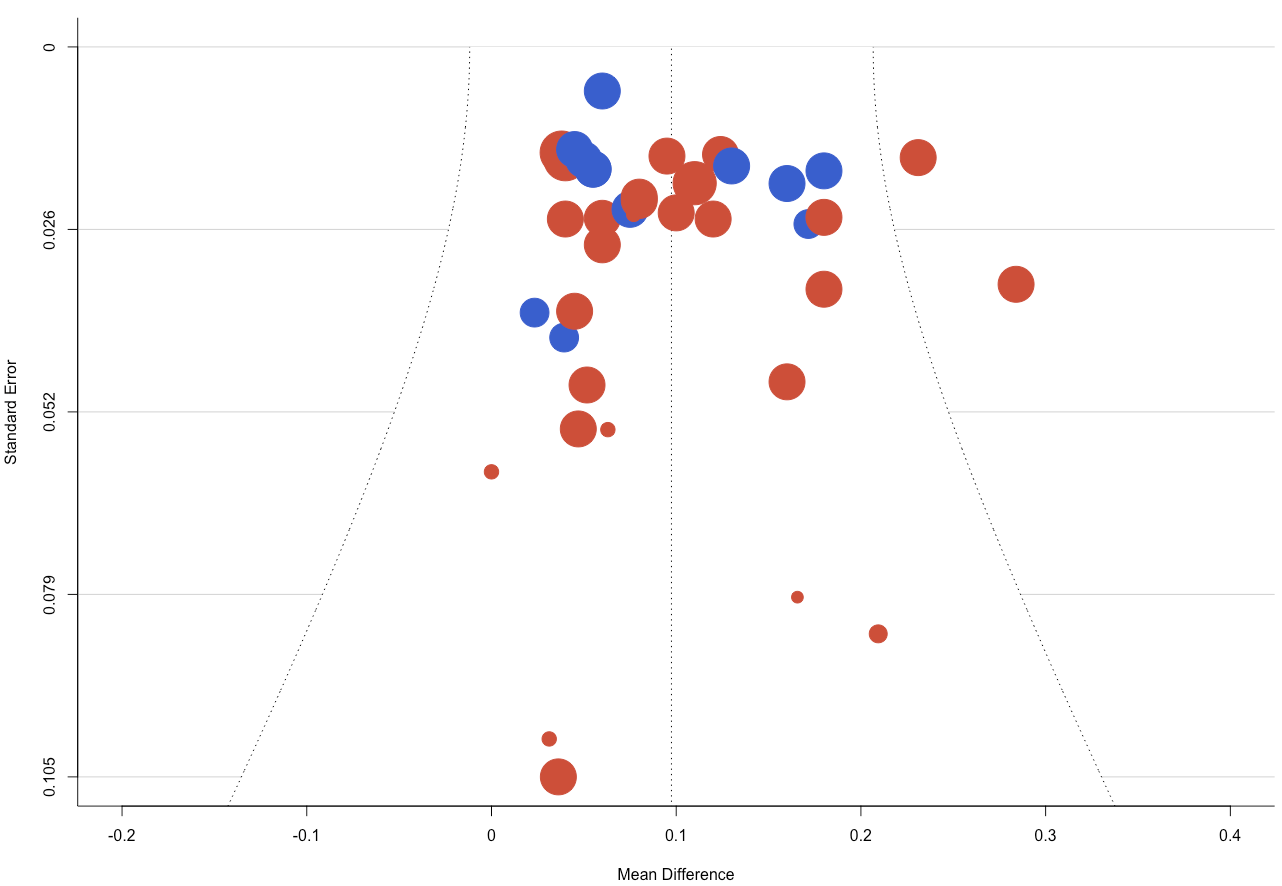


**Appendix 1 – Search Strategy**

Pubmed, Embase and EMCARE were searched using the below strategy

((Diffusion tensor).ti,ab OR (DTI).ti,ab OR (DTI MR).ti,ab OR (Diffusion?tensor).ti,ab OR (DWI DTI).ti,ab OR (DWI).ti,ab OR (Diffusion Weighted).ti,ab OR (Diffusion?Weighted).ti,ab OR (magnetic resonance imaging diffusion tensor).ti,ab OR (Anisotropic diffusion).ti,ab) AND ((Median nerve).ti,ab OR (flexor retinaculum).ti,ab OR (ulnar nerve).ti,ab OR (guyon canal).ti,ab OR (guyon's canal).ti,ab OR (carpal tunnel syndrome).ti,ab OR (median nerve compression).ti,ab OR (entrapment neuropathy).ti,ab OR (nerve entrapment).ti,ab OR (carpal tunnel syndrome).ti,ab OR (CTS).ti,ab OR (neuropathy).ti,ab OR (wrist).ti,ab OR (forearm).ti,ab)

**Appendix 2 – Excluded papers**

1. Meek 2006 – tractography only, no DTI metrics
2. Zhao 2013 (DW MR neurography, no DTI)
3. Allam 2016 – Used 1.5T MRI, no DTI
4. Bao 2016 – Used DW MR neurography, no DTI
5. Bao 2017 – Used DW MR neurography, no DTI
6. Garg 2017 – T1/T2 MRI, no DTI
7. Andreisek 2010 (used same database as Andreisek 2008)
8. Gruggenberger 2012 (agreement between 2 software packages)
9. Zhou 2012 (forearm nerves)
10. Hiltunen 2005 (Peripheral nerves included upper limb and lower limb nerves)
11. Altun 2013 – Relation between electrophysiological findings and diffusion weighted magnetic resonance imaging in ulnar neuropathy at the elbow
12. Breitenseher 2014 – MR neurography of ulnar nerve entrapment at the cubital tunnel: a diffusion tensor imaging study
13. Ding 2016 – Stereoscopic Display of the Peripheral Nerves at the elbow region based on MR diffusion tensor imaging with multiple post-processing methods
14. Iba 2009 - Diffusion-weighted magnetic resonance imaging of the ulnar nerve in cubital tunnel syndrome
15. Jambawalikar 2010 – Diffusion tensor imaging of other peripheral nerves
16. Park 2020 – Determination of optimum pixel size and slice thickness for tractography and ulnar nerve diffusion tensor imaging at the cubital tunnel using 3T MRI
17. Godel 2019 (AIN syndrome)
18. Haakma 2016 (Multifocal motoneuropathy)
19. Kronlage 2017 (Non-specific peripheral nerve imaging)
20. Kronlage 2017(1) (Chronic inflammatory demyelinating polyneuropathy)
21. Karaman 2019 – transection of MN DTI
22. Hsu 2018 – DTI for CTS for steroid injection
23. Baumer 2014 – Peripheral Neuropathy: Detection with diffusion-tensor imaging
